# Serum metabolomic profiling uncovered metabolic shifts in individuals upon moderate-altitude exposure and identified the potentiality of beta-alanine to ameliorate hyperuricemia

**DOI:** 10.1016/j.redox.2025.103546

**Published:** 2025-02-28

**Authors:** Xuanfu Chen, Guoxiang Zou, Zhibo Yang, Xin Qi, Feier Song, Long Peng, Dingchen Wang, Jingyan Zhou, Jiahui Ma, Haiwei He, Yimei Hong, Yu-E Wang, Yanqun Fan, Zhipeng Liu, Xin Li

**Affiliations:** a Guangdong Cardiovascular Institute, Guangdong Provincial People's Hospital (Guangdong Academy of Medical Sciences), Guangzhou, China; b Department of Emergency Medicine, Guangdong Provincial People's Hospital (Guangdong Academy of Medical Sciences), Southern Medical University, Guangzhou, China; c Department of Neurosurgery, Guangdong Provincial People's Hospital Ganzhou Hospital, China; d School of Medicine, South China University of Technology, Guangzhou, China; e The Second Clinical School of Medicine, Southern Medical University, Guangzhou, China; f Nyingchi People's Hospital, Tibet, China; g Biotree Metabolomics Technology Research Center, Shanghai, China

**Keywords:** Moderate altitude, Asymptomatic hyperuricemia, Urate, Serum metabolome, Risk prediction

## Abstract

**Background:**

High-altitude exposure has been associated with an increased risk of hyperuricemia (HU) and gout, though the underlying mechanisms remain poorly understood.

**Methods:**

We conducted a comprehensive analysis of the serum metabolome and phenome in both discovery and validation cohorts of Han Chinese individuals who underwent long-term moderate-altitude exposure (∼12 months), as well as in an independent cohort consisting of local Han Chinese and Tibetans residing in Nyingchi (>5 years). Beta-Alanine intervention was applied in hypoxanthine and potassium oxonate-induced *in vitro* and *in vivo* experiments.

**Results:**

Individuals exposed to moderate altitude exhibited elevated serum urate and an increase in overall medium-chain fatty acids (MCFAs), coupled with a decrease in overall amino acids (AAs) and short-chain fatty acids (SCFAs). Rmcorr correlation analysis revealed a significant negative association between Beta-Alanine and serum urate, whereas nonanoic acid was in versa, potentially driving lower serum urate in long-term exposed residents. Both *in vitro* and *in vivo* experiments demonstrated that Beta-Alanine inhibited xanthine oxidase (XOD) and reversed the HU phenotype in human hepatocytes and mice induced by hypoxanthine (HX) and potassium oxonate (PO), with a urate-lowering effect in mice. Hepatic pathology and transcriptome analysis of HU mice treated with Beta-Alanine indicated that the mechanisms involved the inhibition of XOD, amelioration of the inflammation phenotype in hepatocytes, and promotion of renal urate excretion. Furthermore, the 10-fold cross-validation random forest classification (RFC) predictive modeling based on selected metabolites and phenotypes achieved an area under receiver operating characteristic (ROC) curve (AUC) value of 0.93 (95 % confidence interval (CI): 0.85–1.00) and 0.79 (95 % CI: 0.59–0.98) for distinguishing individuals with high risk of asymptomatic HU (AHU) in the training dataset and validation dataset, respectively.

**Conclusions:**

This study reveals serum urate and metabolome altered in moderate-altitude exposed individuals and Beta-Alanine intervention could ameliorate hyperuricemia. Our findings suggest that targeting the circulating metabolome may pave novel avenues to counter diseases associated with HU.

## Highlights

• The moderate-altitude exposed individuals showed increased serum urate and overall MCFAs, along with decreased overall AAs and SCFAs.
• Beta-Alanine significantly negatively associated with serum urate, while nonanoic acid was in versa, potentially contributing to lower serum urate in long-term exposed residents.
• Beta-Alanine intervention could attenuate hyperuricemia phenome in hypoxanthine and potassium oxonate-induced *in vitro* and *in vivo* experiments.
• The RFC model based on optimal metabolites and phenotypes achieved an AUC of 0.93 (95 % CI: 0.85–1.00) and 0.79 (95 % CI: 0.59–0.98) for distinguishing individuals with high risk of AHU from those with low risk in the training dataset and validation dataset, respectively.

## Graphical abstract

**Figure:**
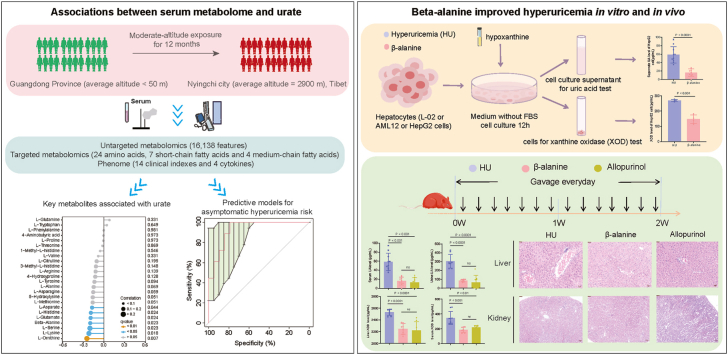

## 1 Introduction

When exploring how the human physiology responds to environmental factors, the impact of high-altitude on metabolism has gained significant interest. High altitude, defined as an elevation above 3000–5500 m, is characterized by low barometric pressure, reduced oxygen levels, temperature variations, and increased ultraviolet radiation [1]. Moderate-altitude exposure, ranging from 2000 to 3000 m [2], poses a unique challenge to the body homeostasis. Previous studies had linked exposure to high altitude to conditions like acute mountain sickness (AMS) [3,4], marked by gastrointestinal maladies [5,6], increased inflammation and heightened susceptibility to illness and infection [[7], [8], [9]]. These physiological changes above 1500 m become more pronounced at altitudes above 3000 m [10,11].

To adapt to these “hostile” conditions, the body employs various mechanisms like increased heart rate, hyperventilation, and an elevated red blood cell count to combat hypobaric hypoxia [12], which induces oxidative stress. Urate contributes to nearly two-thirds of the antioxidant capacity of human blood, produced by XOD, along with the release of free radicals. Accumulating evidences indicated that individuals travelling to high altitude (e.g., 4 weeks at 4560 m or 4 days at 3600 m, followed by 10 days at 5200 m) [13,14], acute exposure to high altitude (e.g., 6 h at 3648 m) or those living at high altitude (e.g., 3640 m, 4100 m or 4200 m) exhibited higher serum or urine urate levels [[15], [16], [17], [18]]. This increase may be due to elevated urate generation resulting from systemic hypoxia and impaired kidney function [19]. Animal studies also demonstrated increased serum urate under high-altitude conditions, such as holstein cows (criollas) and cows descended from fighting bulls (Vacas de lidia) raised at an altitude of 3000 m [20], as well as yak raised at 3000 m, 3500 m, 4000 m and 4300 m [21]. Besides, subjects volunteering for simulated altitude experiments in a hypoxia chamber (e.g., 8 days at 3500 m, 2h at 4500 m, 2h at 3000 m), and Wistar rats raised in a low-pressure oxygen chamber exhibited urate accumulation in their bodies [[22], [23], [24]]. Nevertheless, the underlying biological processes regulating serum urate levels remain incompletely understood due to the complex interplay of genetic, environmental, and lifestyle factors.

Individuals with a fasting serum urate level exceeding 420 μmol/L on two separate days receive a HU diagnosis [25]. HU significantly elevates the risk of developing gout and is linked to various comorbidities, such as chronic kidney disease, diabetes mellitus, hypertension, hyperlipidemia, non-alcoholic fatty liver disease (NAFLD) and cardiovascular diseases [[26], [27], [28]]. Exposure to high altitude can trigger HU. Identifying high-risk individuals empowers them to take preventive measures against HU and its related conditions.

Metabolomics analysis frequently reveals systematic metabolic changes in these conditions, shedding light on the underlying disease-relevant metabolic processes and dysregulation [[29], [30], [31]]. This is accomplished by detecting alterations in the metabolic profile and specific metabolites. Human serum metabolic profiling has pinpointed pathways and metabolites are crucial in regulating serum urate levels at various altitudes, ranging from 50 m to 494 m [[32], [33], [34]]. A consistent observation in these studies is the significant involvement of AAs metabolism in urate regulation. In instances where healthy individuals were exposed to high altitude, such as 4297 m for 14 days, 3500 m for 8 days [22,35], or during an ascent of Cho-Oyu (8201 m) [36], alterations in AAs (e.g., decreased L-Valine, L-Leucine, L-Arginine, L-Citrulline and increased L-Alanine) and purines (e.g., increased hypoxanthine, xanthine and urate) were observed.

However, prior metabolomics studies primarily aimed to uncover potential pathophysiological mechanisms and markers for the progression from AHU to gout in low-altitude regions, or to investigate the effects on host physiology and metabolome profiles in short-term (2 h – 1 month) exposure at altitude exceeding 3000 m. To date, no studies have systematically profiled the longitudinal serum urate and metabolome in human cohorts under moderate-altitude conditions for an extended period (∼12 months) to identify potential associations between serum metabolites and urate. Moreover, no classification models have been established to differentiate or predict the progression from normouricemia (NU) to AHU in individuals exposed to moderate altitude. Understanding how moderate-altitude exposure may predispose individuals to AHU represents a crucial step in unraveling the complex interplay between altitude, metabolism, and health. To delve into this intriguing intersection, our study employed serum metabolomics, a powerful analytical technique offering a comprehensive snapshot of the body's metabolic profile.

In this article, we assessed the impact of moderate-altitude exposure on the serum urate and metabolome of 49 healthy Han Chinese individuals from Guangzhou city (average altitude <50 m) who spent 12 months in Bayi District, Nyingchi city (average altitude = 2900 m) in our discovery cohort. The associations between serum metabolome and urate during the extensive longitudinal profiling were analyzed to reveal key correlations. Besides, the potential beneficial effects of pivotal metabolite on reducing urate were validated *in vitro* and *in vivo*. Additionally, a local cohort of 47 healthy individuals (Han Chinese: Tibetan = 36 : 11) who had resided in Nyingchi (>5 years) was included to tentatively explore the effects of ethnicity and duration of moderate-altitude exposure on serum urate and metabolome. Furthermore, we constructed an RFC model to predict the risk of AHU in individuals following moderate-altitude exposure in the discovery cohort. This model was validated in an independent validation cohort consisting of 47 healthy Han Chinese individuals who resided in Nyingchi for 12 months.

## 2 Methods

### 2.1 Nyingchi city description

Nyingchi city (29.5° N, 94.3° E) covers 80 % of Tibet's total forest area [37], situated alongside the Niyang River, with no heavy industry in the vicinity and tourism serving as the primary economic driver in the region. Detailed meteorological data of Nyingchi city are described in the supplementary methods.

### 2.2 Human study design and samples collection

A total of 143 healthy individuals were recruited for this study, divided into three cohorts:

(1) Discovery cohort (n = 49): This cohort consisted of healthy Han Chinese aid-Tibet volunteers (male/female = 26/23), with an average age of 35.08 ± 6.48 years (mean ± s.d.), who were sent from Guangzhou city to work and stay in Bayi District, Nyingchi city for 12 months starting in May 2018. Anthropometric data and blood samples were collected at baseline, and then at months 0 (week 1), 1, 3, 6, and 12 post-moderate-altitude exposure.
(2) Validation cohort (n = 47): This cohort included healthy Han Chinese aid-Tibet volunteers (male/female = 35/12) from Guangzhou city, with an average age of 40.37 ± 6.06 years (mean ± s.d.), who became residents in Bayi District, Nyingchi city for 12 months starting in July 2018. Anthropometric data and blood samples were collected at baseline, as well as at months 6 and 12 post-moderate-altitude exposure.
(3) Local cohort (n = 47): This cohort comprised healthy volunteers, with two subgroups: the Han Chinese population (Group Local_H) consisting of 36 individuals (male/female = 10/26) with an average age of 30.86 ± 8.30 years (mean ± s.d.), and the Tibetans population (Group Local_T) consisting of 11 individuals (male/female = 1/10) with an average age of 35.73 ± 8.57 years (mean ± s.d.). These individuals had been residing in Nyingchi city over five years. Anthropometric data and blood samples were collected.

The discovery cohort aimed to identify serum metabolites associated with serum urate levels under moderate-altitude exposure. It sought to determine whether these key metabolites and phenotypes could effectively predict serum urate responses in individuals upon one year of moderate-altitude exposure. This was independently validated in the validation cohort. The local cohort was conducted as a pilot study to explore the effects of ethnicity and the duration of moderate-altitude exposure on serum urate and metabolome. Throughout the follow-up, some healthy Han Chinese aid-Tibet volunteers left Nyingchi due to work arrangements and dropped out. All eligible subjects were screened for disease and underwent physical examinations according to the 1999 WHO criteria. None of the study subjects had taken antibiotics, anti-obesity agents, hormones, or probiotics for at least one month before the study began. The study protocol was approved by the Human Research and Ethics Committee of the People's Hospital of Nyingchi and was registered at ChiCTR.org.cn (*ChiCTR1800016854*), according to the principle of the Helsinki Declaration II. Everyone gave written informed consent.

**Fig. 1:**
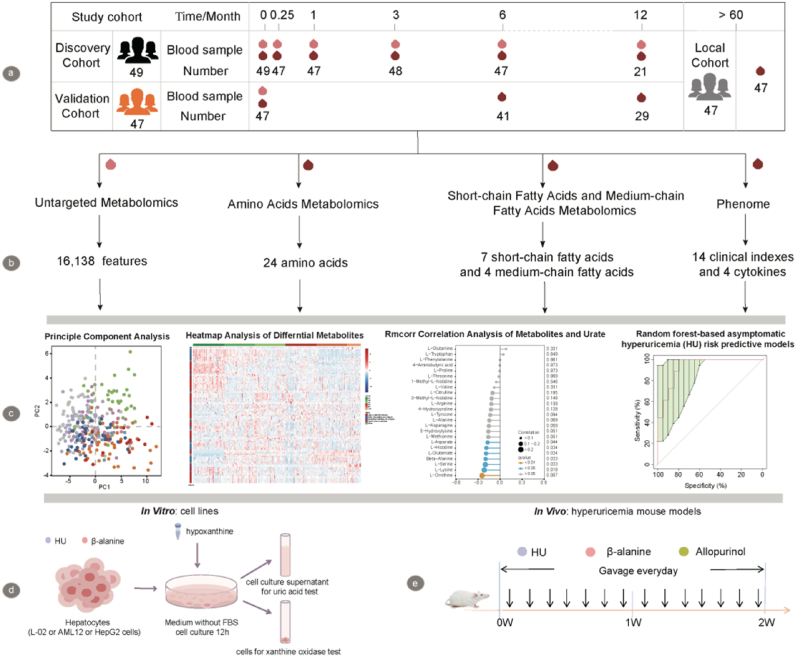
Overview of the workflows integrating host untargeted serum metabolomics, targeted serum metabolomics and phenome in this study. Untargeted serum metabolomics technique was performed to discover significantly differential metabolites in individuals upon moderate-altitude exposure, which were further validated with targeted amino acids measurement and replenished with targeted short-chain fatty acids and medium-chain fatty acids detection. Functional metabolites ameliorating urate were subsequently identified and validated *in vitro* and *in vivo*, and diagnostic models for identifying individuals at high risk of progressing to asymptomatic hyperuricemia during moderate-altitude exposure were constructed.

Venous blood samples for biochemical measurements were collected following an overnight fast (>10 h). Serum samples were centrifuged and stored at −80 °C until analysis. In total, 259 serum samples from the discovery cohort and 47 serum samples from the validation cohort underwent untargeted metabolomics analysis, and 423 serum samples from the discovery cohort (259), validation cohort (117), and local cohort (47) were used for quantifying AAs, SCFAs, and MCFAs (Fig. 1).

### 2.3 Cell culture and treatments

Primary hepatocytes, including human liver cells (L-02) and mouse liver cells (AML12), were purchased from BeNa Culture Collection (BNCC; China), while hepatocellular carcinoma cells (HepG2) were procured from the American Type Culture Collection (ATCC; USA). L-02 and AML12 cells were cultured in Dulbecco's Modified Eagle Medium/Nutrient Mixture F-12 (DMEM/F-12; Gibco, 11320033), and HepG2 cells were maintained in Dulbecco's minimal essential medium (DMEM; Gibco, 11965092). All cell cultures supplemented with 10 % fetal bovine serum (FBS; HyClone, SV3020802), 100 U/mL penicillin, and 100 μg/mL streptomycin (Thermo Fisher Scientific, A5873601). Cells were incubated at 37 °C in a humidified atmosphere containing 5 % carbon dioxide (CO_2_). After 72 h, when cell confluence reached approximately 80–90 %, the cultures were rinsed with phosphate-buffered saline (PBS), treated with trypsin, and reseeded at a density of 6∼8 × 10^5^ cells per well in a 6-well plate containing the appropriate medium without FBS. After a further 24 h of incubation, L-02 and AML12 cells were exposed to 1000 μM hypoxanthine for 12 h, while HepG2 cells were treated with 150 μM hypoxanthine for 12 h [38,39]. The cells were randomly assigned to one of two groups: the HU group and the Beta-Alanine group. Prior research has demonstrated that Beta-Alanine supplementation is safe at concentrations of 3 % [40]. Therefore, the Beta-Alanine group was provided with a medium containing 3 % Beta-Alanine. Subsequent assays were performed to quantify urate levels in the supernatant (Boxbio, AKAO014 M), and the cells were collected for measurement of XOD (Jianglai, JL20379 for mice and JL19475 for human).

### 2.4 Mouse study and samples collection

Male Kunming mice (18–20 g, 4 weeks old) were obtained from the Guangdong Medical Laboratory Animal Center. The mice were housed in a Specific Pathogen-Free (SPF) experimental animal facility, maintained at a controlled temperature of 23 ± 2 °C and a humidity of 60 ± 10 %, under a strict 12-h light/dark cycle. The mice were acclimated for one week before the experiment, during which they had unrestricted access to standard food and water.

The mice were randomly assigned to one of six groups: (a) the healthy control group, (b) the hyperuricemia group (HU), (c) the Beta-Alanine treatment group (with concentrations of 1 %, 2 % or 3 % Beta-Alanine in drinking water), and (d) the allopurinol treatment group (administered at a dosage of 5 mg/kg body weight), with each group comprising ten animals. The dosages of Beta-Alanine and allopurinol utilized in this study were established based on existing literature [[41], [42], [43]]. In terms of *in vivo* administration, meta-analyses indicate that oral Beta-Alanine supplementation in murine models is safe at daily doses of up to 3 % [40]. Based on this safety threshold, we categorized Beta-Alanine supplementation into low, medium, and high concentrations, with 3 % as the highest concentration.

To induce HU, all experimental groups, except the healthy control group, were subjected to daily oral gavage of PO at a dose of 900 mg/kg (Macklin, P831461) and HX at a dose of 500 mg/kg (Macklin, H811076) for a duration of two weeks [[43], [44], [45]]. These compounds were suspended in a 0.5 % carboxymethyl cellulose sodium (CMC-Na) saline solution. Throughout this treatment period, the HU and Allopurinol groups were provided with standard drinking water, whereas the Beta-Alanine group received drinking water containing 1 %, 2 % or 3 % Beta-Alanine (Macklin, A6018) [41,42]. Additionally, the Allopurinol group was administered 5 mg/kg of allopurinol (Macklin, A800424) via oral gavage 1 h after PO and HX administration.

Body weights were recorded every other day. On the 14th day, 2 h after the final treatment, the mice were anesthetized with 0.03 mg/g pentobarbital. Blood was collected from the orbital plexus, centrifuged at 3000 rpm for 15 min at 4 °C, and the serum was separated and stored at −80 °C for subsequent analysis. The liver and kidneys were dissected and stored at −80 °C for subsequent analysis. Transcriptome sequencing was performed on the right lobe of the liver. Urate levels (Boxbio, AKAO014 M) were measured in serum and urine. XOD levels (Jianglai, JL20379-96T) was accessed in serum and liver tissue. Hematoxylin-eosin (H&E) staining was performed on liver and kidney tissues. mRNA was extracted from these tissues, and RT-qPCR was used to conduct mRNA expression level of different genes, all the primers sequences were provided in Table S13. Further details are provided in the supplementary methods.

All procedures were approved by the Research Ethics Committee of Guangdong Provincial People's Hospital, Guangdong Academy of Medical Sciences with the approval number: *KY-N-2022-010-02*.

### 2.5 Human biochemical parameters and cytokines measurements

Anthropometric data, such as height, body weight (BW), waist circumference (WC), body mass index (BMI), heart rate (HR) and blood pressure (systolic blood pressure (SBP) and diastolic blood pressure (DBP)), were obtained using standard protocols. Routine blood tests including serum lipid profiles (total cholesterol (CHOL), high-density lipoprotein cholesterol (HDL), and low-density lipoprotein cholesterol (LDL), triglycerides (TG)), serum urate, serum creatinine (SCr), fast blood glucose (FBG) and blood urea nitrogen (BUN), were assessed using standard laboratory techniques with a mindray BS600 Analyzer. Serum leptin and adiponectin levels were detected with ELISA kits purchased from Elabsicence.cn and Guangdong Uniten Biotechnology Co., Ltd., respectively, according to the manufacturer's instructions. Serum insulin levels were measured by chemiluminescence immunoassay using Atellica IM1200 analyzer.

The concentrations of serum *IL-1β*, *IL-6*, *IL-10* and *TNF-α* were assessed using ELISA kits from Elabscience. Detection was performed with Multiskan FC (Thermo Fisher Scientific) following the manufacturer's instructions. Additionally, the levels of Superoxide Dismutase (SOD) and Glutathione Peroxidase (GPX) in the serum were quantified using Boxbio kits (AKAO001M and AKPR014M).

### 2.6 Untargeted serum metabolome and targeted serum metabolome acquisition

*Untargeted metabolome profiles.* Serum samples were preprocessed with modifications as previously described [46]. LC-MS/MS analyses were conducted utilizing an ultra-high performance liquid chromatography (UHPLC) 1290 system (Agilent Technologies, Waldbronn, Germany) coupled with a UPLC HSS T3 column (2.1 mm × 100 mm, 1.8 μm, Waters, Manchester, UK) interfaced with a Q Exactive mass spectrometer (Orbitrap MS, Thermo Fisher Scientific, SanJose, CA). Details are described in the supplementary methods.

*Targeted AAs profiles.* Serum AAs quantification was performed as previously described with modifications [47]. LC-MS/MS analyses were conducted through a Agilent 1290 Infinity II series UHPLC System (Agilent Technologies) equipped with a Waters ACQUITY UPLC BEH Amide column (100 × 2.1 mm, 1.7 μm, Waters) interfaced with a Agilent 6460 triple quadrupole mass spectrometer (Agilent Technologies) equipped with an AJS electrospray ionization (AJS-ESI) interface. Details are described in the supplementary methods.

*Targeted SCFAs and MCFAs profiles.* Serum SCFAs and MCFAs quantification were performed as previously described [48] and analyzed using SHIMADZU GC2030-QP2020 NX gas chromatography-mass spectrometer (GC-MS) (Shimadzu Corporation, Kyoto, Japan) equipped with a HP-FFAP capillary column (30 m × 250μm × 0.25 μm, Agilent Technologies, Wilmington, DE, USA). Details are described in the supplementary methods.

### 2.7 Untargeted metabolomics data analysis

A three-dimensional matrix containing arbitrarily assigned peak indices (retention time - m/z pairs), ion intensities (variables) and sample names (observations) were acquired as previously described with slight modifications [31]. Details are described in the supplementary methods.

### 2.8 Mouse liver transcriptome sequencing

Total RNA was extracted from the tissue using TRIzol® Reagent according to the manufacturer's instructions. The high-quality RNA samples were subsequent for the paired-end RNA-seq library construction, which was sequenced on the NovaSeq X Plus sequencer (2 × 150 bp read length) (Illumina, San Diego, CA). Details are described in the supplementary methods.

### 2.9 RNA sequencing data analysis

To identify differentially expressed genes (DEGs) between samples, transcript expression levels were calculated using the transcripts per million (TPM) method. Additionally, functional enrichment analyses, including Gene Ontology (GO) and Kyoto Encyclopedia of Genes and Genomes (KEGG) pathways, were performed. Details are described in the supplementary methods.

### 2.10 Rmcorr correlation analysis

Rmcorr method was applied for association analysis of metabolites and serum urate within each subject alongside moderate-altitude exposure [49]. *P*-values were corrected by p.adjust (method = “fdr”) in R.

### 2.11 Metabolome and phenome-based classifier

The baseline NU individuals with serum urate level exceeding 420 μmol/L after moderate-altitude exposure were classified as high risk of AHU (AHU-HR) group, and the baseline NU individuals with normal serum urate concentrations after moderate-altitude exposure were classified as low risk of AHU (AHU-LR) group. The RFC models were trained in the discovery cohort and then externally evaluated on in the validation cohort. The analysis involved performing 5 repetitions of 10-fold cross-validation. We used cross-validation error curves to select features, following the approach described by Ren et al. [29]. The cross-validation error curves from the 5 trials were averaged, and the minimum error in the averaged curve plus the standard deviation at that point served as the cutoff for an acceptable error. From the group of features with a classification error below the cutoff, we selected the set with the fewest features as the optimal set [50]. The risk probability of AHU-HR for each subject was computed for each dataset, and the AUC was calculated. Furthermore, the phenotypic prediction model and the combinations of metabolic and phenotypic prediction model were constructed in the same way.

### 2.12 Statistical analysis

Unless otherwise stated, all statistical analyses were performed in the R software (v3.6.2), and *P*-values after false discovery rate correction less than 0.05 were considered as statistically significant level. *P*-values for categorical data were calculated with Fisher's exact test. *P*-values for discontinuous data were calculated by chi-square test. *P*-values of paired or unpaired samples were calculated with paired or unpaired two-tailed Wilcoxon test, respectively. *P*-values were adjusted by the Benjamini-Hochberg correction for multiple tests when required. Targeted metabolome profiles were analyzed by principal component analysis (PCA) with SIMCA 16 (Umeå, Sweden). The relative changes of metabolites over time were investigated by linear mixed model (LME) using the ‘lme4’ package (v1.1). Significant pathways were identified with metabolomics pathway analysis (MetPA) tools in MetaboAnalyst [51].

## 3 Results

### 3.1 The effects of moderate-altitude exposure on serum urate levels

**Fig. 2:**
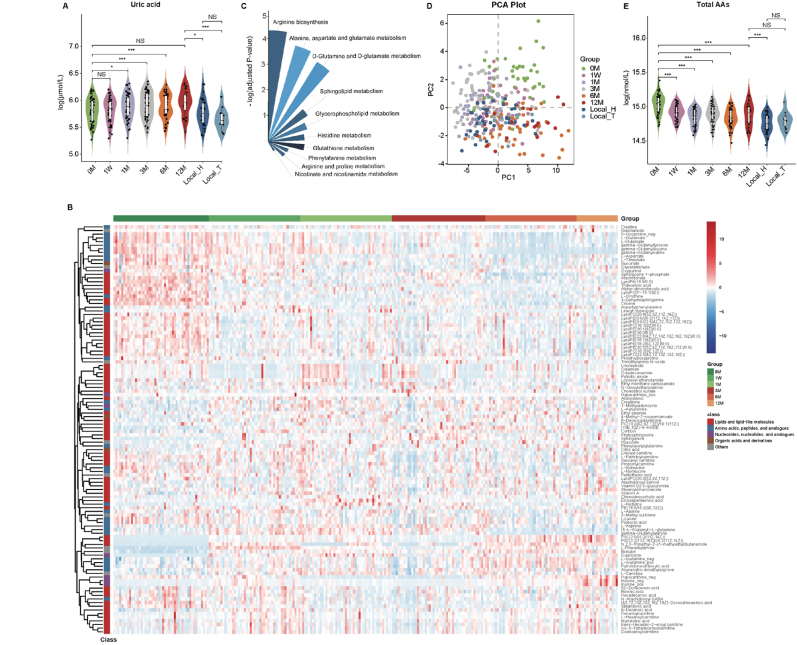
Serum urate levels, untargeted serum metabolome and targeted serum amino acids (AAs) profiles in the discovery and local cohorts. **A** Violin plot of serum urate levels. Plotted are interquartile ranges (IQRs; boxes), medians (dark lines in the boxes), the lowest and highest values within 1.5 times IQR from the first and third quartiles (lines above and below the boxes), and density of values (width between curves). P-value of paired/unpaired samples was calculated with paired/unpaired two-tailed wilcox test. ∗*P* < 0.05; ∗∗*P* < 0.01; ∗∗∗*P* < 0.001; NS, not significant. **B** Hierarchical cluster analysis of 112 significantly differential metabolites (adjusted *P* < 0.05). **C** Rose diagram of the differential metabolism pathways revealed by metabolic pathway analysis (MetPA). Each slide represents one pathway with pathway impact value > 0, and the size of each slide is based on -log(adjusted *P*). **D** The principal component analysis (PCA) scores plot for targeted serum amino acids profiles in the discovery and local cohorts. **E** The serum concentrations of total AAs in the discovery and local cohorts, and violin plot as in Fig. 1A.

An abnormal elevation of blood urate levels is a hallmark of HU. In the discovery cohort, we observed a significant increase in serum urate levels correlated with the duration of moderate-altitude exposure, as shown by the LME model (adjusted *P* = 0.008; Fig. 2A; Table S1). This finding aligns with previous reports indicating that high-altitude exposure leads to higher serum urate concentrations. However, Han Chinese and Tibetans residing in Nyingchi (>5 years) exhibited significantly lower serum urate levels compared to the 12 M group (*P* = 0.014, 0.003, respectively; Fig. 2A). Furthermore, the serum urate levels in the 12 M group became statistically insignificant compared to the baseline, although they still displayed an increasing trend. These phenomena were consistent in the validation cohort (Additional file 3: Table S1). These findings suggested that the human body might become more accustomed to moderate-altitude exposure over time.

### 3.2 The effects of moderate-altitude exposure on serum amino acids

To explore changes in the serum metabolome during moderate-altitude exposure, we previously conducted untargeted metabolomics analysis on 259 serum samples in the discovery cohort. The analysis revealed that the relative standard deviation of internal standards was low, indicating minimal variability during detection. Moreover, there was no observable drift in the quality control samples across the metabolome profiles (Additional file 2: Fig. S1B), suggesting that the metabolome data exhibited good stability and reproducibility. Using the LME model, we identified 112 significant metabolites with significant alterations in abundance during moderate-altitude exposure (adjusted *P* < 0.05; Fig. 2B; Additional file 3: Table S2). Additionally, MetPA analysis revealed significant pathways alterations in individuals exposed to moderate altitudes. Notably, Arginine biosynthesis, Alanine, Aspartate, and Glutamate metabolism, D-Glutamine, and D-Glutamate metabolism showed substantial changes (adjusted *P* = 0.01, 0.02, 0.02, respectively, with pathway impact value > 0) (Fig. 2C; Additional file 3: Table S3). These findings indicated noteworthy alterations in AAs among individuals exposed to moderate altitude.

We then validated the observed differences in individuals exposed to moderate altitude by measuring the levels of 24 circulating AAs in 306 serum samples from both the discovery and local cohorts. The PCA scores plot illustrated significant alterations in circulating AAs profiles in those exposed to moderate altitude (Fig. 2D). Overall AAs levels were decreased in these individuals (Fig. 2E). Importantly, all AAs changes depended on the duration of moderate-altitude exposure, as indicated by the LME model (adjusted *P* < 0.05; Additional file 3: Table S4). Specifically, in the Arginine biosynthesis pathway, concentrations of L-Arginine, L-Ornithine, L-Citrulline, L-Glutamate, and L-Asparate decreased, while L-Glutamine increased in individuals exposed to moderate altitude. Changes in other AAs associated with L-Arginine metabolism were also observed, including decreased L-Proline and 4-Hydroxyproline in the Arginine and Proline metabolism, increased L-Alanine but decreased L-Asparagine in the Alanine, Aspartate, and Glutamate metabolism of individuals exposed to moderate altitude (Additional file 2: Fig. S1C). Moreover, the serum AAs profiles of individuals exposed to moderate altitude became like those of individuals residing in Nyingchi (>5 years) (Fig. 2D–E).

### 3.3 The effects of moderate-altitude exposure on serum short-chain fatty acids and medium-chain fatty acids

**Fig. 3:**
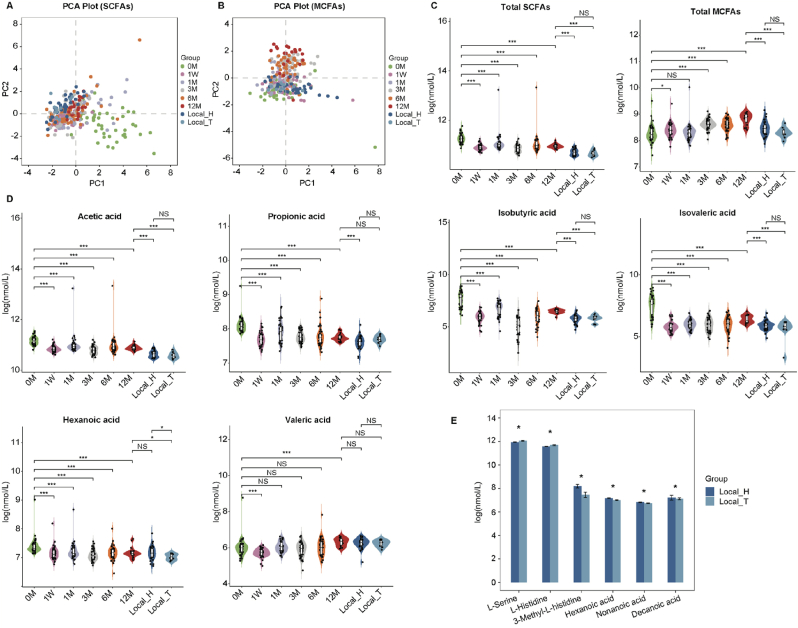
Targeted serum short-chain fatty acids (SCFAs) and medium-chain fatty acids (MCFAs) in the discovery and local cohorts. **A** The principal component analysis (PCA) scores plot for targeted serum SCFAs profiles in the discovery and local cohorts. **B** PCA scores plot for targeted serum MCFAs profiles in the discovery and local cohorts. **C** Violin plot of serum overall SCFAs and MCFAs in the discovery and local cohorts, plotted as in Fig. 1A. **D** Specifically significantly altered SCFAs and MCFAs in the healthy individuals exposed to moderate altitude, plotted as in Fig. 1A. **E** Four metabolites differed between the Local_H group and the Local_T group. Data were expressed as mean ± SE. Significance between every two groups was calculated using unpaired two-tailed wilcox test. ∗*P* < 0.05; ∗∗*P* < 0.01; ∗∗∗*P* < 0.001.

**Fig. 4:**
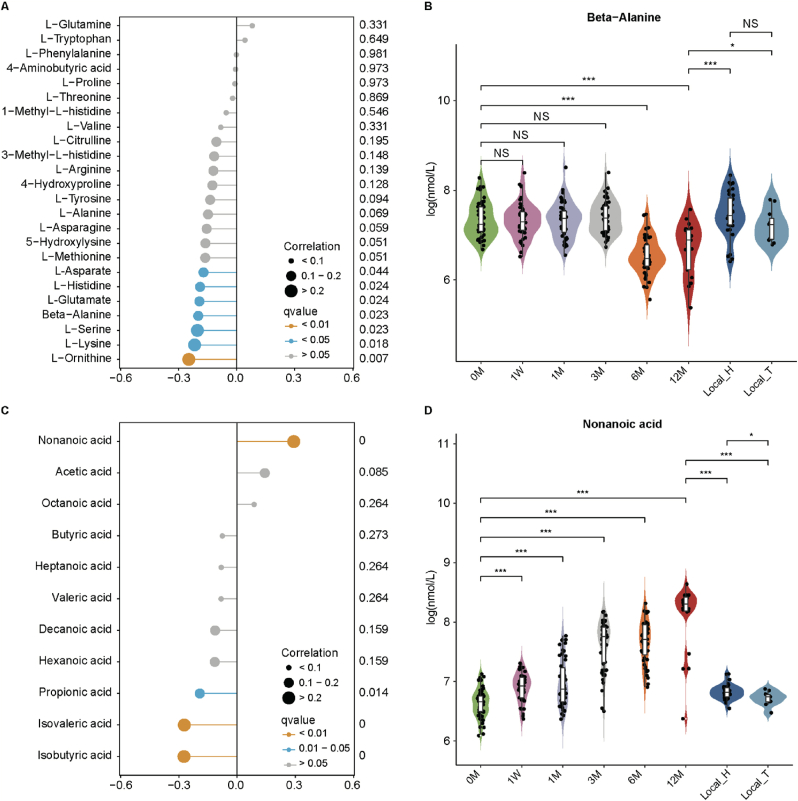
Rmcorr correlation analysis of serum urate and targeted serum metabolites. (**A, C**), lollipop plot of the Rmcorr correlation coefficients between serum urate and amino acids (AAs) (**A**), between serum urate, short-chain fatty acids (SCFAs) and medium-chain fatty acids (MCFAs) in the discovery cohort (**C**), respectively. The size and color of each circle are based on Rmcorr correlation coefficients and adjusted Rmcorr *P*-value, respectively. (**B, D**), violin plot of serum Beta-Alanine (**B**) and nonanoic acid (**D**) levels in the discovery and local cohorts, plotted as in Fig. 1A.

Previous metabolomic studies had highlighted strong correlations between gut microbial-derived metabolites, endogenous host metabolites, and various diseases [52]. Colonic gut microbiota could ferment the microbial-accessible carbohydrates and proteins into the precursors for the biosynthesis of SCFAs and MCFAs [53]. We further measured 7 SCFAs and 4 MCFAs in 306 serum samples from the discovery and local cohorts to investigate the shift in serum metabolome associated with moderate-altitude exposure. PCA scores plot demonstrated significant changes in serum SCFAs and MCFAs profiles in individuals undergoing moderate-altitude exposure (Fig. 3A and B). The total SCFAs decreased while overall MCFAs increased in individuals exposed to moderate altitude (Fig. 3C). Except for heptanoic acid, other SCFAs and MCFAs changed depending on the duration of moderate-altitude exposure, as revealed by the LME model (adjusted *P* < 0.05; Additional file 3: Table S5). Specifically, acetic acid, propionic acid, isobutyric acid, isovaleric acid, and hexanoic acid significantly decreased, while valeric acid and nonanoic acid significantly increased in individuals exposed to moderate altitude (Fig. 3, Fig. 4D). Furthermore, we observed that the serum SCFAs and MCFAs profiles of individuals exposed to moderate altitude resembled those of individuals residing in Nyingchi (>5 years) (Fig. 3A and B).

### 3.4 Comparison of serum urate and metabolome profiles between Han Chinese populations and Tibetans residing in Nyingchi (>5 years)

As a pilot study, we examined the impact of ethnicity on serum urate and metabolome profiles in Tibetans and Han Chinese populations residing in Nyingchi (>5 years). No significant difference in serum urate levels was observed between the Local_H and Local_T groups (*P* = 0.17, Fig. 2A). Among the 24 detected AAs, 7 detected SCFAs, and 4 detected MCFAs, 4 metabolites (3-Methyl-L-histidine, hexanoic acid, nonanoic acid and decanoic acid) were higher and 2 metabolites (L-Serine and L-Histidine) were lower in the Han Chinese populations than the Tibetan populations (*P* = 0.02, 0.04, 0.02, 0.04, 0.04, 0.02, respectively, Fig. 3E). These data suggested only minor differences in serum AAs, SCFAs, and MCFAs profiles between the Local_H and Local_T groups. Given the limited sample size and measured metabolites, further research is needed to explore the longitudinal and ethnic effects on serum urate and metabolome profiles in Tibetans in the future.

### 3.5 Associations analysis of serum amino acids, short-chain fatty acids, medium-chain fatty acids and urate

To identify correlations between serum AAs, SCFAs, MCFAs and urate levels in individuals upon moderate-altitude exposure, we performed Rmcorr correlation analysis. Our findings revealed that L-Ornithine, L-Lysine, L-Serine, Beta-Alanine, L-Glutamate, L-Histidine and L-Asparate exhibited strong negative associations with urate (Rmcorr adjusted *P* < 0.05; Fig. 4A). No significant positive correlations between AAs and urate were observed. As previously noted, serum urate concentrations were markedly lower in Han Chinese and Tibetan populations residing in Nyingchi (>5 years) compared to the 12 M group. This observation led to the hypothesis that the relationship between urate and AAs might differ in these populations, potentially contributing to the reduced serum urate levels seen in the Nyingchi residents. Among these 7 AAs negatively correlated with urate, Beta-Alanine was the only one to exhibit a significant increase in individuals living in Nyingchi (>5 years) when compared to the 12 M group (Fig. 4B; Additional file 2: Fig. S2A). Furthermore, isobutyric acid, isovaleric acid, and propionic acid demonstrated strong negative correlations with urate, whereas nonanoic acid showed the opposite pattern (Rmcorr adjusted *P* < 0.05; Fig. 4C). Interestingly, nonanoic acid was found to be significantly lower in individuals residing in Nyingchi (>5 years) compared to the 12 M group (Fig. 4D; Additional file 2: Fig. S2B). Besides, correlation analysis between Beta-Alanine and fatty acids indicated that Beta-Alanine exhibited a significant negative correlation solely with nonanoic acid, with no significant associations observed with other fatty acids (Additional file 2: Fig. S2C). Taken together, these findings implied that higher Beta-Alanine or lower nonanoic acid might be beneficial for individuals accustoming to moderate-altitude conditions, potentially influencing urate metabolism.

### 3.6 The effects of moderate-altitude exposure on oxidative stress responses

Prior research has established a correlation between high-altitude exposure and oxidative stress, particularly in acute high-altitude conditions [54,55]. Superoxide Dismutase (SOD) is a crucial metal-containing enzyme that plays a vital role in the antioxidant defense system by neutralizing reactive oxygen species (ROS). In contrast, Glutathione Peroxidase (GPX) catalyzes the oxidation of glutathione (GSH) to its oxidized form (GSSG), thereby converting toxic hydrogen peroxide into less harmful hydroxyl compounds [56]. As shown in Figs. S2D–E, we observed that short-term exposure to moderate altitude resulted in decreased levels of SOD and GPX. However, these levels gradually returned to baseline values typically seen at sea level as individuals acclimatized. These trends were inversely related to urate levels (Fig. 2A), which contributed to nearly two-thirds of the antioxidant capacity in human blood.

Additionally, serum metabolomic profiles were altered during the acclimatization process, with the profiles of individuals exposed to moderate altitude becoming more similar to those of long-term residents (Fig. 2D). Notably, Beta-Alanine levels decreased in the moderate-altitude group during the 12-month longitudinal survey, while the residents, who had lower urate levels, showed an increase in Beta-Alanine (Fig. 4B). These findings collectively suggested that individuals undergoing moderate-altitude exposure adapted to the environment by dynamically reprogramming their metabolism, including responses to oxidative stress, changes in the serum metabolome, and regulation of urate levels. Moreover, γ-glutamyl amino acid metabolites, formed via the extracellular membrane-bound enzyme γ-glutamyltransferase, facilitated the transfer of a γ-glutamyl moiety from glutathione to amino acids and peptides in response to ROS with glutamate production as a byproduct. L-glutamine, a metabolite known for its protective role against hypoxic stress, as well as its importance in maintaining the intestinal barrier, immune function, and microbial diversity, was also examined [57,58]. As illustrated in Fig. 2B, we observed a consistent reduction in L-glutamate and its associated dipeptides (e.g., γ-glutamylleucine, γ-glutamylvaline, γ-glutamylalanine, γ-glutamyltyrosine), while L-glutamine levels increased monotonically in individuals exposed to moderate altitude, which again suggested a metabolic adaptation to hypoxic stress within the host system.

### 3.7 Beta-alanine attenuated urate accumulation in hypoxanthine and potassium oxonate-induced *in vitro* and *in vivo* experiments

**Fig. 5:**
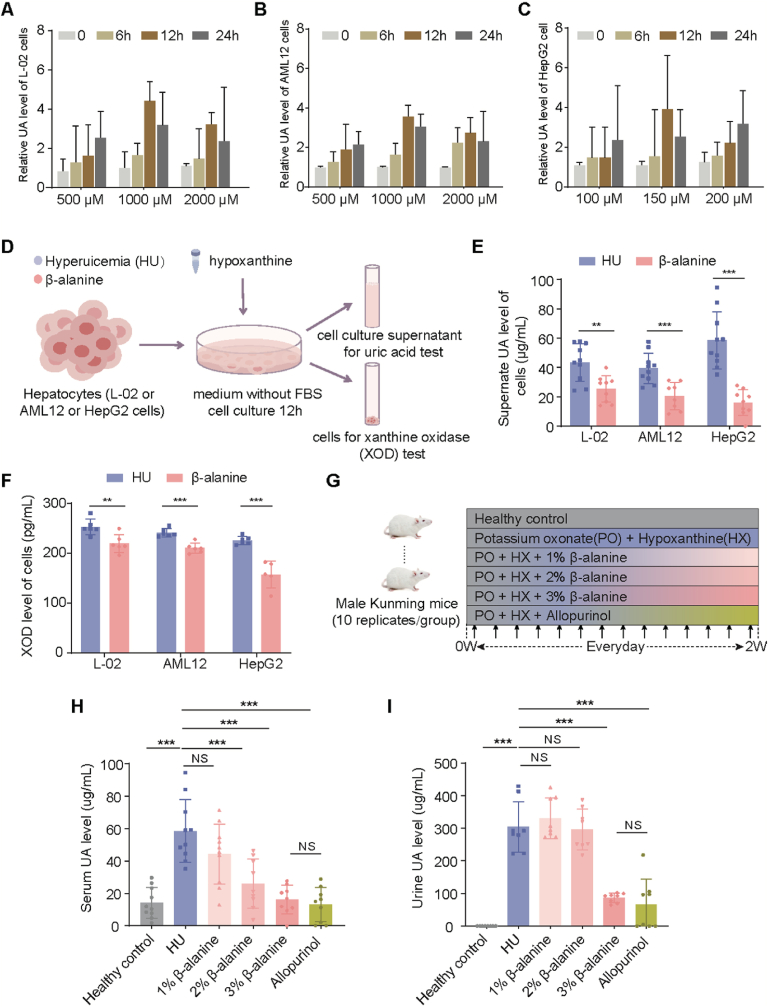
Beta-Alanine reduced urate levels both *in vitro* and *in vivo* models. **A-C** Optimization of hypoxanthine concentration and incubation time *in vitro* experiments. **D***In vitro* experimental design. **E-F** The urate (UA) and xanthine oxidase (XOD) levels in human primary hepatocytes (L-02 cells), mouse liver cells (AML12 cells) and hepatocellular carcinoma cells (HepG2 cells). Each spot represents one replicate. Experiment was conducted with 9–10 replicates **E** or 5–6 replicates **F** per group. **G***In vivo* experimental design. **H–I** Serum and urine urate levels. Experiment was conducted with 8–10 samples per group. Data are shown as mean ± SEM. Each spot in figure **E**, **F**, **H** and **I** represents one sample. Significance between every two groups was calculated using unpaired two-tailed Student's t-test. ∗*P* < 0.05; ∗∗*P* < 0.01; ∗∗∗*P* < 0.001; NS, not significant.

To evaluate the efficacy of Beta-Alanine in ameliorating HU phenome, we established HU cell models using either human primary hepatocytes (L-02 cells) or hepatocellular carcinoma cells (HepG2 cells) and mouse liver cells (AML12 cells), both treated with hypoxanthine for 12h (Fig. 5A–D). And Beta-Alanine was administrated accordingly. Differences in urate metabolism might exist between hepatocellular carcinoma cells and normal hepatocytes, requiring varied hypoxanthine concentrations (150 μM vs 1000 μM). Notably, Beta-Alanine significantly reduced urate synthesis and XOD expression in all HU cell models (Fig. 5E–F). Subsequently, a hyperuricemia mouse model was developed using PO and HX (Fig. 5G). By the end of modeling, HU mice treated with Beta-Alanine or allopurinol had lower blood and urine urate levels (Fig. 5H–I). All the HU and intervention groups showed higher excretion of urate compared with that of the healthy control group. Besides, administration of 1 % Beta-alanine resulted in higher urinary urate excretion when compared to other groups. However, increasing the concentration of Beta-Alanine further led to a reduction in urinary urate levels, which might be attributed to the decreased blood urate concentrations, potentially counteracting the renal excretion effect.

**Fig. 6:**
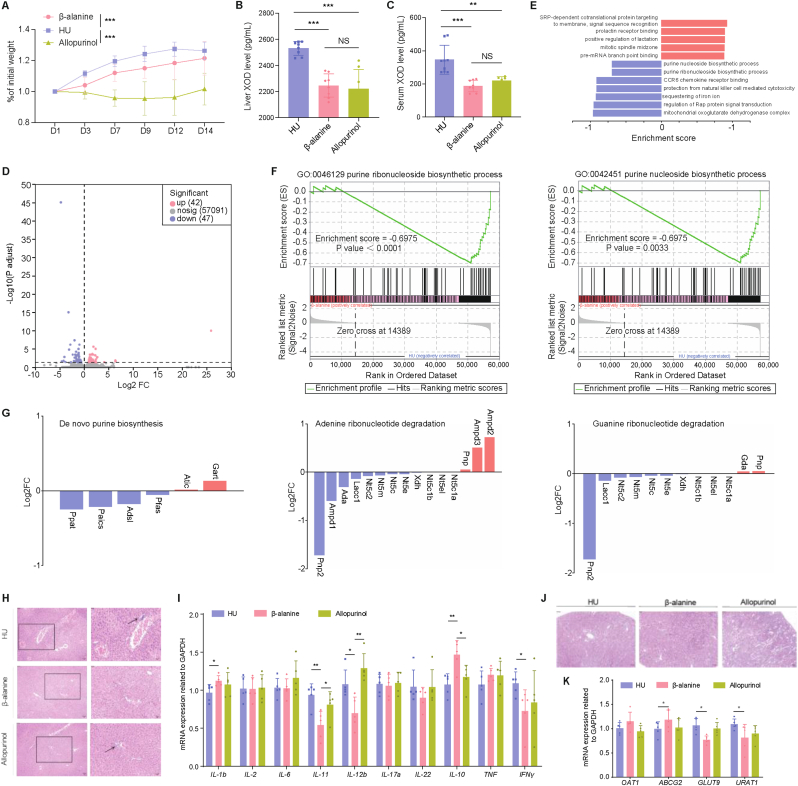
Beta-Alanine ameliorated hyperuricemia (HU) associated physiology and affected inflammation and urate metabolism associated genes expression *in vivo* models. **A** Body-weight gain. **B–C** Liver and serum XOD levels. **D** Volcano plot showing differentially expressed genes in the hepatic tissue between HU mice treated with Beta-Alanine (n = 4) and HU mice (n = 4). Genes with |log2FC (Beta-Alanine/HU)| ≥ 1 and adjusted *P* < 0.05 are marked in red or blue for upregulated and downregulated genes in HU treated with Beta-Alanine mice vs. HU mice, respectively. **E** Significantly altered pathways revealed by gene set enrichment analysis (GSEA) based on Gene Ontology (GO) database in hepatic tissue (*P* < 0.05). **F** GSEA results of purine ribonucleoside biosynthetic process pathway and purine nucleoside biosynthetic process in hepatic tissue. **G** Genes expression levels in the pathways of de novo purine biosynthesis, adenine ribonucleotide degradation and guanine ribonucleotide degradation in hepatic tissue. **H** Representative images of H&E staining of hepatic tissue. Scale bars, 20 μm. **I** mRNA levels of inflammatory factor genes in the hepatic tissue. **J** Representative images of H&E staining of renal tissue. Scale bars, 20 μm. **K** mRNA levels of UA excretion correlated genes in the renal tissue. Data are shown as mean ± SEM. Each spot in figure **B**, **C**, **I** and **K** represents one sample. Experiment was conducted with 7–9 samples (**B, C**) or 5 samples (**I, K**) per group. In figure **A**, significance between every two groups was calculated using two-way repeated-measures ANOVA. In figure **B**, **C**, **I** and **K**, significance between every two groups was calculated using unpaired two-tailed Student's t-test. ∗*P* < 0.05; ∗∗*P* < 0.01; ∗∗∗*P* < 0.001; NS, not significant.

Compared with 1 % and 2 % Beta-alanine intervention in mice HU model, the 3 % Beta-alanine showed the most significant reduction in blood and urine urate levels (Fig. 5H–I). Hence, the 3 % Beta-alanine was selected for subsequent experiments. Over the course of the intervention, body weight of HU group mice increased more significantly (Fig. 6A). Furthermore, analysis of the liver, the primary organ responsible for urate production, revealed that XOD expression in both the liver and serum of HU mice treated with Beta-Alanine was reduced, similar to the levels observed in the allopurinol-treated group (Fig. 6B–C). The results of transcriptome sequencing and GSEA analysis showed that purine metabolism pathway was less active in HU mice treated with Beta-Alanine compared to untreated HU mice, e.g. the genes expression levels of de novo purine synthesis pathway, and adenine and guanine nucleotide degradation pathway were lower than those in HU mice (Fig. 6D–G; Additional file 3: Tables S6–S7). HE staining of liver tissues revealed obvious inflammations in both HU and allopurinol-treated mice than the Beta-Alanine group (Fig. 6H). These groups also exhibited higher expression levels of pro-inflammatory factors, including *IL-11*, *IL-12b*, and *IFN-γ*, compared to Beta-Alanine-treated mice, whereas the expression of the anti-inflammatory factor *IL-10* was lower in the HU and allopurinol groups than in the Beta-Alanine group (Fig. 6I). However, we also observed that *IL-1β* significantly increased in the Beta-Alanine group, and there were much more pro-inflammatory factors with increased tendency in the allopurinol group, which deserved further investigation in the future. Renal pathology in the HU and allopurinol groups showed basement membrane thickening, partial glomerular sclerosis, and thickening and hyaline degeneration of the renal arterioles (Fig. 6J). We further assessed the expression of well-known renal urate metabolism related genes. Our findings revealed that the expression of ABCG2 (ATP-binding cassette subfamily G member 2), a urate efflux transporter, was higher in the Beta-Alanine group than in the HU group (Fig. 6K). Conversely, the expression of GLUT9 (glucose transporter 9) and URAT1 (urate transporter 1), mediating urate reabsorption, was lower in the Beta-Alanine group compared to the HU group. However, GLUT9 and URAT1 showed a decreasing trend in the allopurinol group, with no significant difference compared to the model group. These results indicated a potential enhancement of urate excretion by Beta-Alanine intervention in HU mice.

### 3.8 Pilot study of the metabolic and phenotypic prediction model to predict the asymptomatic hyperuricemia response of normouricemia individuals upon moderate-altitude exposure

**Fig. 7:**
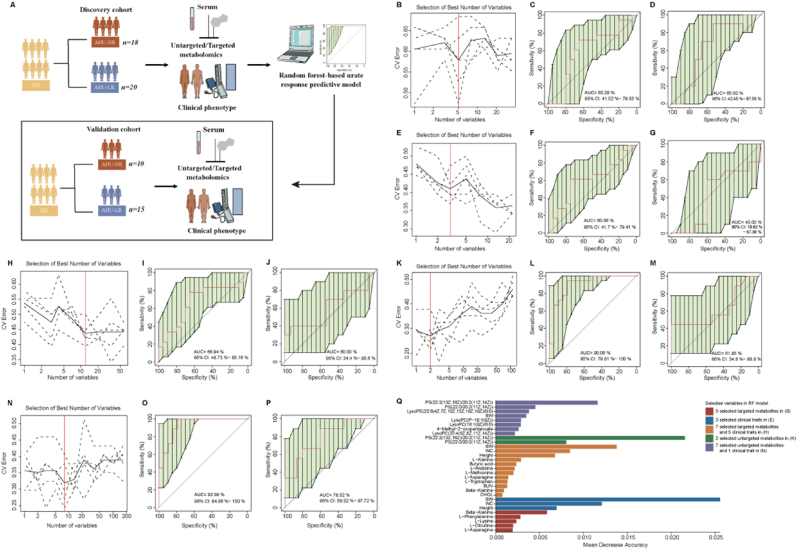
Prediction performance of serum metabolome and clinical phenome for asymptomatic hyperuricemia response. **A** Workflow demonstrating the construction of prediction models and compositions of the asymptomatic hyperuricemia (AHU) response phenotype in the normouricemia (NU) individuals upon moderate-altitude exposure in the discovery and validation cohorts. (**B, E, H, K, N**), distribution of 5 trials of 10-fold cross-validation error in random forest classification (RFC) models using targeted serum metabolites (**B**), clinical phenotype traits (**E**), combinations of targeted serum metabolites and clinical phenotype traits (**H**), untargeted serum metabolites (**K**), combinations of untargeted/targeted serum metabolites and clinical phenotype traits (**N**), respectively. The model was trained with clinical factors in the training set (high risk of AHU (AHU-HR) group, n = 18; low risk of AHU (AHU-LR) group, n = 20). The black solid curve showed the average of the 5 trials (dash lines). The red line indicated the number of picked features in the optimal set. (**C, F, I, L, O**), the area under the receiver operating characteristic (ROC) curve (AUC) of 5 selected targeted metabolites (**C**), 3 selected clinical traits (**F**), 7 selected targeted metabolites and 5 clinical traits (**I**), 2 selected untargeted metabolites (**L**), 7 selected untargeted metabolites and 1 clinical trait (**O**) for the discovery cohort, respectively. (**D, G, J, M, P**), the ROC curve and area under the ROC curve in the validation cohort with AHU-HR subjects (n = 10) and AHU-LR subjects (n = 15) by respective RFC model in **C**, **F**, **I**, **L**, **O**. (**Q**) The importance measured by mean decrease accuracy of the selected features in the above models between AHU-HR group and AHU-LR group in the discovery and validation cohorts. BW, body weight; WC, waist circumference; BUN, blood urea nitrogen; CHOL, total cholesterol.

Exposure to moderate altitude increases serum urate levels, putting individuals at risk of AHU. Predicting serum urate responses in individuals exposed to moderate altitude could be beneficial for implementing interventions to prevent the development of AHU. To investigate the predictive potential of AHU response in NU individuals upon moderate-altitude exposure, we excluded baseline samples with AHU and samples collected after the initiation of moderate-altitude exposure. This focus was on serum metabolome and phenome for urate response prediction. We categorized individuals into two groups in the baseline of discovery and validation cohorts: AHU-HR group and AHU-LR group (Fig. 7A).

A 10-fold cross-validation RFC model was used to select the optimal feature panels to investigate whether metabolic profiling or clinic profiling could predict future AHU development in NU subjects upon moderate-altitude exposure. The targeted metabolic model, containing 5 features (i.e., Beta-Alanine, L-Phenylalanine, L-Lysine, L-Citrulline and L-Asparagine) achieved an AUC of 0.60 (95 % CI: 0.41–0.80) and 0.65 (95 % CI: 0.42–0.88) for predicting responders and non-responders in the training dataset and validation dataset, respectively (Fig. 7B–D; Additional file 3: Table S8). The phenotypic model containing 3 features (i.e., BW, WC and height) achieved an AUC of 0.61 (95 % CI: 0.42–0.79) and 0.43 (95 % CI: 0.19–0.68) in the training dataset and validation dataset, respectively (Fig. 7E–G; Additional file 3: Table S9). The combined targeted metabolic and phenotypic model, containing 12 features (i.e., Beta-Alanine, L-Alanine, L-Histidine, L-Methionine, L-Asparagine, L-Tryptophan, butyric acid, BW, WC, height, BUN and CHOL) achieved an AUC of 0.67 (95 % CI: 0.49–0.85) and 0.60 (95 % CI: 0.34–0.86) in the training dataset and validation dataset, respectively (Fig. 7H–J; Additional file 3: Table S10). Combinations of targeted serum metabolome and phenome enhanced the predictiveness of serum urate response, indicating the involvement of metabolites in serum urate regulations. Beta-Alanine were selected as one of these vital metabolites by above-mentioned RFC models, which again suggested Beta-Alanine was important in the urate metabolism homeostasis regulations.

Then, we tried to investigate whether other metabolites would enhance the predictiveness of serum urate response with those 112 significant metabolites discovered by untargeted serum metabolome technology. Interestingly, the untargeted metabolic model, containing 2 features (i.e., phosphatidylserine (PS) (22:2(13Z,16Z)/20:2(11Z,14Z)) and PS(22:0/20:2(11Z,14Z))) achieved an AUC of 0.90 (95 % CI: 0.80–1.00) and 0.62 (95 % CI: 0.35–0.89) in the training dataset and validation dataset, respectively (Fig. 7K–M; Additional file 3: Table S11), which were superior than the above-mentioned models. We further evaluated the potential importances of PS(22:2(13Z,16Z)/20:2(11Z,14Z)) and PS(22:0/20:2(11Z,14Z) by combining untargeted serum metabolome, targeted serum metabolome and phenome together to establish combined prediction model. The combined metabolic and phenotypic model containing 8 features achieved an AUC of 0.93 (95 % CI: 0.85–1.00) and 0.79 (95 % CI: 0.59–0.98) in the training dataset and validation dataset, respectively (Fig. 7N–P; Additional file 3: Table S12). Among these 8 features, PS(22:2(13Z,16Z)/20:2(11Z,14Z)) and PS(22:0/20:2(11Z,14Z) ranked as the top 2 important metabolites. These demonstrated that except of Beta-Alanine, PS(22:2(13Z,16Z)/20:2(11Z,14Z)) and PS(22:0/20:2(11Z,14Z) might play pivotal roles in serum urate metabolism regulations, warranting research in the future. All the selected features in the above-mentioned models were shown in Fig. 7Q. These preliminary results suggested it was possible to distinguish NU individuals with AHU-HR response upon moderate-altitude exposure by serum metabolome profiles.

## 4 Discussion

Previous research had linked elevated serum or urine urate levels to high-altitude exposure [[13], [14], [15], [16], [17]]. Animal studies indicated that different animals experienced increased serum urate levels at varying high altitude [20,21]. However, limited knowledge existed about individuals at risk of AHU during moderate-altitude exposure. For the first time, we profiled three independent cohorts with varying durations and ethnic backgrounds based on a comprehensive serum metabolome, including untargeted and targeted metabolites components such as AAs, SCFAs, and MCFAs. This allowed for a comprehensive comparison of linked serum metabolites with urate, an assessment of the effects of ethnicity and the duration of moderate-altitude exposure on the host metabolome and phenotype, and the development of a predictive model to identify individuals at high risk of AHU upon moderate-altitude exposure. And we further validated the potentiality of Beta-Alanine in ameliorating high urate phenome in HX and PO induced human hepatocytes and mice, indicating a new therapy for preventing or counteracting HU.

Serum urate levels in long-term residents of Nyingchi, encompassing both Han Chinese and Tibetan populations, were notably lower compared to individuals who had been in the region for only one year. This finding aligned with previous research that populations residing in the Tibetan Plateau (altitude >4300 m) exhibited notably lower UA levels than those living on the plains (altitude <1700 m) [59]. These observations suggested that prolonged exposure to moderate-altitude environments might trigger physiological adaptations that resulted in lower serum urate concentrations. In our study, levels of SOD and GPX decreased upon initial exposure to moderate altitude, but exhibited a dynamic increase during the acclimatization process. This pattern contrasted with the observed trends in urate levels. Furthermore, serum metabolomic profiles were altered during acclimatization, with individuals exposed to moderate altitude showing metabolomic patterns similar to those of long-term residents. Notably, Beta-Alanine levels decreased in individuals exposed to moderate altitude over the 12-month longitudinal survey, while levels increased in residents with lower urate concentrations. Taken together, these findings suggested that prolonged adaptation to moderate-altitude environments involves a dynamic reorganization of metabolism, oxidative stress responses, serum metabolome, and urate levels.

Supporting this notion, earlier studies demonstrated that individuals, including passengers and crewmembers who spent 8.5 h in a hypobaric chamber simulating an altitude of 2438 m [60], followed by additional exposure to a simulated altitude of 3658 m, exhibited a decrease in serum urate levels at the higher altitude. Furthermore, individuals with lower urate levels and elevated neutrophil counts appeared to demonstrate greater resistance to acute mountain sickness during acute high-altitude exposure at 3648 m [15]. A recent study involving three independent cohorts further substantiated these findings [61]. In this study, cohorts included a longitudinal cohort of 46 healthy male adults who traveled from Chongqing (a.s.l. 243 m) to Lhasa (a.s.l. 3658 m) and back over a period of 108 days, as well as two long-term exposure cohorts consisting of 163 Han residents living at high altitudes for 5–60 months and 28 native Tibetans from Shigatse (a.s.l. 4700 m). The study observed an initial increase in intestinal urate levels upon hypoxia exposure, followed by a reduction during acclimatization. Notably, the decrease in intestinal urate levels during acclimatization was coincided with an increase in urate-degrading bacteria, supporting the hypothesis that reduced urate levels might be an adaptative response to high-altitude hypoxia. Additionally, pre-exposure to simulated high altitude may facilitate acclimatization and provide a potential novel strategy for managing serum urate levels during exposure to high-altitude exposure. In experimental studies, rats pre-exposed to simulated altitudes of 5000 m and 6000 m for 2 h per day 2–11 days achieved acclimatization to hypoxia at a simulated altitude of 8000 m [62]. This acclimatization was characterized by increased mitochondrial glutamate dehydrogenase activity, ketone bodies formation, and reduced lactate and urate production. In skeletal muscle of mice and human, high altitude exposure rewired the TCA cycle for the decreased O2 delivery and increased L-Glutamine to protect against hypoxia stress [57,63]. In the liver, serum and brain of mice, as well as isolated primary murine hepatocytes, tryptophan associated kynurenine synthesis faded, while tryptamine production enhanced following hypoxia exposure [64]. These metabolic adjustments in response to hypoxia were observed in multiple organs, with the heart shifting to increased glucose oxidation, while the brain, kidney and liver showed enhanced fatty acid uptake and oxidation along with reductions in blood glucose levels and adiposity [65]. In line with these reported findings, our study observed a similar trajectory of urate levels, increased L-Glutamine while reduced fasting blood glucose, BMI and fatty acids in moderate-altitude exposed individuals, which collectively indicated these volunteers underwent physiological acclimatization to moderate-altitude environment. However, the specific mechanisms through which the human body (e.g., the respiratory, cardiovascular, and hematological systems) adapted to these challenging conditions remained unknown and could involve gene selection and human adaptation [12,66,67], which were warranted for further research.

Besides, layers of evidences reported that gut microbiota were engaged in the metabolism of urate, for example, the urate catabolism phylum *Proteobacteria*, the genera *Alistipes*, *Bifidobacterium* and *Lactobacillus*, the species *Alistipes indistinctus* (*A*. *indistinctus*), *Alistipes putredinis* (*A*. *putredinis*) and *Eubacterium ramulus*, the urate biosynthesis species *Klebsiella pneumoniae* (*K*. *pneumoniae*) [59,[68], [69], [70], [71]]. We reanalyzed our metagenome data of healthy individuals before and after moderate-altitude exposure in another cohort [72] and found *K*. *pneumoniae* showed decreased trend while *Alistipes*, *A*. *indistinctus* and *A*. *putredinis* showed increased trend in individuals upon moderate-altitude exposure (Additional file 2: Fig. S3A). *Proteobacteria*, *Bifidobacterium*, *Lactobacillus* and *Eubacterium ramulus* decreased in 12M group and turned to increase during acclimatization. These indicated the imbalance of urate metabolism engaged gut microbiota together with serum urate kindly restored balanced again during acclimatization. Moreover, we explored whether the above discovered microbiota had the capability in the synthesis or catabolism of Beta-Alanine. The catabolism of Beta-Alanine to anti-hyperuricemic pantothenate by K01918 (pantoate-beta-alanine ligase [EC:6.3.2.1]), associated with increased *A*. *putredinis*, was upregulated (Additional file 2: Fig. S3B) [73,74]. These indicated that increased urate catabolism microbiota (e.g., *A*. *putredinis*) and decreased urate biosynthesis microbiota (e.g., *K*. *pneumoniae*) might participate in the homeostasis metabolism of urate in moderate-exposed individuals, contributing to the lowered urate in residents.

Untargeted serum metabolomics revealed that Arginine biosynthesis was the most significantly altered metabolism in individuals exposed to moderate altitude. Further validation through targeted AAs profiles confirmed significant decreases in L-Arginine, L-Ornithine, L-Citrulline, L-Glutamate, L-Asparate, L-Proline, and 4-Hydroxyproline. Prior studies had noted decreased levels of L-Arginine, L-Citrulline, and L-Glutamate, along with increased urate, xanthine, and hypoxanthine in subjects exposed to high altitudes, such as 3500 m for 8 days or 4297 m for 14 days [22,35], or acute hypoxic conditions with 12 % and 15 % O_2_ (equivalent to approximately 4500 m and 3000 m in altitude) for 2 h in a normobaric hypoxia chamber [23]. These short-term hypoxia exposures resulted in changes to AAs and purine metabolism similar to those seen in long-term moderate-altitude exposure, suggesting that L-Arginine and urate could potentially serve as markers for hypoxic conditions. Besides, several studies had reported decreased blood arginine levels in individuals with HU-associated conditions like gout and knee osteoarthritis [32,75], reduced L-Arginine transport and biosynthesis in hyperuricemic rats, mouse liver and human L02 hepatocytes, and adult rat ventricular cardiomyocytes upon urate stimulus [76,77]. Both acute and chronic L-Arginine treatment prevented glomerular hypertension and preglomerular arteriolopathy in male Sprague-Dawley rats with HU [78]. These findings suggested that L-Arginine might play a crucial role in the progression of HU and be a therapy for HU-associated diseases. However, it's important to note that these studies were not conducted in high-altitude environments. Therefore, further research is needed to explore the mechanisms of L-Arginine in HU-associated disorders and to determine whether L-Arginine could be an effective treatment for preventing HU under high-altitude conditions.

Apart from the reduced overall AAs, we also observed a decrease in overall SCFAs but an increase in overall MCFAs in individuals exposed to moderate altitudes. However, residents in Nyingchi showed significantly lower overall MCFAs. In addition, isobutyric acid, isovaleric acid and propionic acid were significantly negatively associated with serum urate, while nonanoic acid was in versa. Branched-chain fatty acids (BCFA) (e.g., isobutyric acid and isovaleric acid) are a class of SCFAs produced in the gut through the proteolytic fermentation of branched-chain amino acids. Previous studies had demonstrated that BCFA may play a pathologic role in cardiometabolic disease by blocking lipolysis and elevating glucose production [[79], [80], [81], [82], [83], [84]]. The negative correlations between circulating isobutyric acid, isovaleric acid, and urate further suggested that urate contributes to the antioxidant and protective role in human blood. Propionic acid, as one of the main SCFAs produced in the gut. The role of propionic acid in the metabolic phenotype has been debated. It is related to overweight, obesity and a higher risk of type 2 diabetes [72,[85], [86], [87], [88]]. Conversely, propionic acid can attenuate atherosclerosis [89], multiple sclerosis disease [90], autism [91], hemodialysis [92], nonalcoholic steatohepatitis and improves cerebrovascular functions [93]. These indicated that propionic acid exhibited complex and diverse functions depending on the disorder, and its negative relationship with urate warrants further research. Nonanoic acid, a medium-chain fatty acid, could induce the thymic stromal lymphopoietin production and exacerbate allergic inflammation in mice [94]. It increased in Sjögren's syndrome [95], induced irritant contact dermatitis [96], inhibited the penetration of biotin through the blood-brain barrier [97], and induced epidermal langerhans cell apoptosis *in vivo* [98]. These suggested that nonanoic acid could be considered a chemical allergo-accelerators and might play a role in promoting elevated urate in individuals exposed to moderate altitude. The underlying mechanisms regarding the regulatory effects of how nonanoic acid interacted with Beta-Alanine in urate metabolism deserved further investigation in the future.

Furthermore, residents in Nyingchi (>5 years) exhibited lower serum urate levels, accompanied by a significantly increase in Beta-Alanine and a decrease in nonanoic acid compared to individuals exposed to moderate-altitudes for 12 months. Other AAs, SCFAs or MCFAs maintained a consistent trend without reversal during more extended moderate-altitude exposure. Besides, Beta-Alanine showed a negative association with urate, contrasting nonanoic acid. Beta-alanine, a nonessential amino acid synthesized in the liver, combined with histidine to form the intracellular dipeptide carnosine, a crucial intracellular buffer [99,100]. Beta-alanine had been shown to elevate carnosine in the brain and muscle [101], enhanced anaerobic skeletal muscle performance, which benefited exercise capacity [102], and increased resilience to post-traumatic stress disorder, mild traumatic brain injury, and heat stress [[103], [114]]. Despite its widespread use by strength/power athletes, studies on the effects of Beta-Alanine supplementation on serum urate remained scarce. Our findings suggested that Beta-Alanine exerted protective effects by reducing urate levels through the inhibition of XOD in the *in vitro* and *in vivo* models.

Moreover, Beta-Alanine intervention demonstrated systemic effects, including reducing weight gain in hyperuricemia mice and ameliorating inflammation phenotype. This was evidenced by reduced inflammations in the liver tissue and lower expression of pro-inflammatory factors like *IL-11*, *IL-12b*, and *IFN-γ*. Simultaneously, there was an increase in the expression of the anti-inflammatory factor *IL-10*, which helped mitigate liver inflammations [104,105]. While in Beta-Alanine intervened HU mice, *IL-1β* expression increased and *TNF-α* levels showed a trend toward elevation without reaching statistical significance. Our review of the literatures highlighted that *IL-1β*, although primarily associated with pro-inflammatory effects, also played a role in cell phenotype transformation and neuroprotection. And the allopurinol intervention in HU mice resulted in much widely increased tendency in inflammatory markers (*IL-1β*, *IL-6*, *IL-11*, *IL-12b*, *IL-22*, *TNF-α*, *IFN-γ*) than that of Beta-Alanine intervention. The inflammatory process, including its initiation and resolution, is complex and may involve regulatory pathways that are not yet fully understood. Additionally, Beta-alanine reduces renal inflammations, decreases urate reabsorption, and promotes renal urate excretion in HU mice, which may slow the progression of hyperuricemic kidney disease [106,107]. The effect of allopurinol on the kidney is still debated. However, as an external drug, it may still cause kidney damage [[108], [109], [110]]. These findings suggested that Beta-Alanine, as a safe supplement, could potentially be used in conjunction with other drugs to treat hyperuricemia, enhancing therapeutic efficacy while reducing drug-related toxic side effects. Whether Beta-Alanine had better amelioration effects on inflammation and promoted anti-inflammation effects than allopurinol should be further explored in the future, and these mechanisms warranted further investigation.

AHU is strongly linked to various disease conditions. Therefore, it is necessary to construct prediction models to assess the risk of AHU before moderate-altitude exposure in NU individuals. Our pilot study showed the targeted metabolic and phenotypic model showed AUC values of AUC of 0.67 (95 % CI: 0.49–0.85) and 0.61 (95 % CI: 0.37–0.85), and the untargeted metabolic and phenotypic model had AUC values of 0.93 (95 % CI: 0.85–1.00) and 0.78 (95 % CI: 0.59–0.97) for predicting responders and non-responders in the training dataset and validation dataset, respectively. These models offered a potentially new approach to diagnose and prevent AHU with minimal adverse effects. In spite of Beta-Alanine being selected as pivotal metabolite, PS(22:2(13Z,16Z)/20:2(11Z,14Z)) and PS(22:0/20:2(11Z,14Z)) were screened out as top 2 metabolites in RFC models. PS levels were positively correlated with urate, but negatively correlated with glomerular filtration rate [111,112]. Consistently, we found PS(22:2(13Z,16Z)/20:2(11Z,14Z)) and PS(22:0/20:2(11Z,14Z)) significantly increased in individuals upon moderate-altitude exposure. These findings suggested that, apart from AAs, phospholipids metabolism may play a pivotal role in regulating serum urate.

While our study suggested promising and potentially applicable connections between the serum metabolome and host urate, we recognized several limitations. First, since all participants were of Chinese ethnicity, the findings may not generalize to other ethnic groups, much more independent cohorts with larger population, longer durations and larger sample sizes were warranted. Second, apart from AAs, SCFAs, and MCFAs, further research is needed to determine if additional bioactive metabolites influence serum urate levels. Third, our population-based analysis results should be interpreted with caution, as unmeasured factors like lifestyle and genetics may have impacted our findings. Fourth, the underlying mechanisms of Beta-Alanine in reducing urate, and its potential applications in AHU or HU individuals should be further investigated. We hope our findings inspire the development of novel therapeutic strategies for diseases associated with HU and encourage future experimental and clinical studies to provide more mechanistic insights into the relationship between moderate-altitude exposure and HU-related disorders.

## 5 Conclusions

In summary, our findings extend our insights into the relationships among the moderate-altitude exposure, serum urate and host metabolome. The identified metabolites, Beta-Alanine, which could reduce serum urate in the *in vitro* and *in vivo* experiments, may be considered for future HU intervention therapies. The prediction model, based on serum metabolites and phenotypes, demonstrated the potential to identify NU individuals at high risk of AHU during moderate-altitude exposure. This will foreseeably have a profound impact on clinical assessment and management, helping to counteract the progression of HU and other comorbidities.

## CRediT authorship contribution statement

**Xuanfu Chen:** Formal analysis, Investigation, Methodology, Project administration, Visualization, Writing – original draft, Writing – review & editing. **Guoxiang Zou:** Investigation, Project administration, Supervision. **Zhibo Yang:** Investigation, Project administration, Supervision. **Xin Qi:** Investigation, Project administration, Supervision. **Feier Song:** Investigation, Project administration, Supervision. **Long Peng:** Investigation, Project administration, Supervision. **Dingchen Wang:** Investigation, Project administration, Supervision. **Jingyan Zhou:** Investigation, Project administration, Supervision. **Jiahui Ma:** Investigation, Project administration, Supervision. **Haiwei He:** Investigation, Project administration, Supervision. **Yimei Hong:** Investigation, Project administration, Supervision. **Yu-E Wang:** Investigation, Project administration, Resources, Supervision. **Yanqun Fan:** Conceptualization, Data curation, Investigation, Methodology, Project administration, Software, Visualization, Writing – original draft, Writing – review & editing. **Zhipeng Liu:** Conceptualization, Investigation, Project administration, Supervision, Writing – original draft, Writing – review & editing. **Xin Li:** Conceptualization, Funding acquisition, Resources, Supervision, Writing – original draft, Writing – review & editing.

## Availability of data and materials

Metabolomics data were uploaded to Metabolights (accession numbers: MTBL1809 and MTBLS9081). Data analysis in this study mainly relied on open-source XCMS for untargeted metabolomics, and mentioned tools used for the data analysis were applied with default parameters unless specified otherwise.

## Ethics approval and consent to participate

The human study protocol was approved by the Human Research and Ethics Committee of the People's Hospital of Nyingchi and was registered at ChiCTR.org.cn (*ChiCTR1800016854*), according to the principle of the Helsinki Declaration II. Everyone gave written informed consent. The animal study protocol was approved by the Research Ethics Committee of Guangdong Provincial People's Hospital, Guangdong Academy of Medical Sciences with the approval number: *KY-N-2022-010-02*.

## Consent for publication

All authors approved the final manuscript before submission.

## Funding

The study work was supported by grants from the National Key Research and Development Program Intergovernmental Key Projects (No. 2023YFE0114300), the Joint Funds of the Natural Science Foundation of China (No. U24A20652), National Science Foundation of China (No. 82272246), Basic and Applied Basic Research Foundation of Guangdong Province (No. 2024A1515012697), Science and Technology Program of Guangzhou (No. 202206010044), High-level Hospital Construction Project of Guangdong Provincial People’s Hospital (No. DFJHBF202104).

## Declaration of competing interest

The authors declare that they have no known competing financial interests or personal relationships that could have appeared to influence the work reported in this paper.

## Appendix A Supplementary data

Multimedia component 2 Multimedia component 2

Fig. S1 Quality control analysis in serum metabolome measurement and an atlas of L-Arginine engaged metabolism pathways associated with the healthy individuals exposed to moderate altitude. **A** The relative standard deviations of internal standards in untargeted metabolome and targeted metabolome (amino acids, AAs; short-chain fatty acids, SCFAs; medium-chain fatty acids, MCFAs) analysis. **B** The principal component analysis (PCA) scores plot for quality control (QC) samples in untargeted metabolomics profile. **C** Amino acids (AAs) associated with L-Arginine metabolism, such as Arginine biosynthesis pathway, Arginine and Proline metabolism, and Alanine, Aspartate, and Glutamate metabolism, were colored with grey. Plotted are interquartile ranges (IQRs; boxes), medians (dark lines in the boxes), the lowest and highest values within 1.5 times IQR from the first and third quartiles (lines above and below the boxes), and density of values (width between curves). P-value of paired/unpaired samples was calculated with paired/unpaired two-tailed wilcox test. ∗*P* < 0.05; ∗∗*P* < 0.01; ∗∗∗*P* < 0.001; NS, not significant. Fig. S1

Fig. S2 Trajectories of metabolites significantly associated with serum urate and correlation analysis between Beta-Alanine and fatty acids, and oxidative stress indexes measurements. **A** Boxplot of amino acids significantly correlated with serum urate. Plotted are interquartile ranges (IQRs; boxes), medians (dark lines in the boxes), the lowest and highest values within 1.5 times IQR from the first and third quartiles (lines above and below the boxes), and outliers beyond the lines (circles). **B** Boxplot of short-chain fatty acids and medium-chain fatty acids significantly correlated with serum urate. Plotted as A. **C** Rmcorr correlation analysis of Beta-Alanine and fatty acids. The size and color of each circle are based on Rmcorr correlation coefficients and adjusted Rmcorr *P*-value, respectively. **D-E** Superoxide Dismutase (SOD) and Glutathione Peroxidase (GPX) levels in individuals before and after moderate-altitude exposure. Data were expressed as mean ± SE. *P*-value of paired/unpaired samples was calculated with paired/unpaired two-tailed wilcox test. ∗*P* < 0.05; ∗∗*P* < 0.01; ∗∗∗*P* < 0.001. Fig. S2

Fig. S3 Violin plot of literatures reported gut microbiota engaged in the metabolism of urate in another cohort. **A** Relative abundance of gut microbiota at phylum, genus and species levels related to urate metabolism. **B** Relative abundance of K01918 in the metabolism of Beta-Alanine to anti-hyperuricemic anserine. Plotted are interquartile ranges (IQRs; boxes), medians (dark lines in the boxes), the lowest and highest values within 1.5 times IQR from the first and third quartiles (lines above and below the boxes), and density of values (width between curves). *P*-value of paired/unpaired samples was calculated with paired/unpaired two-tailed wilcox test. ∗∗*P* < 0.01; ∗*P* < 0.05; +*P* < 0.1; NS, not significant. Fig. S3
