# Supplementary material for: Serum metabolomic profiling uncovered metabolic shifts in individuals upon moderate-altitude exposure and identified the potentiality of beta-alanine to ameliorate hyperuricemia
Source: Redox Biol. 2025 Feb 28;81:103546. doi: 10.1016/j.redox.2025.103546 (PMC11930757; doi:10.1016/j.redox.2025.103546)
Supplement: Multimedia component 1 [file mmc1.docx]

**Supplementary Methods**

**Site description**

Nyingchi city (29.5° N, 94.3° E), also known as "Little Switzerland" in China, is situated along the Niyang River, a major tributary of the Brahmaputra. Located in the southeastern Qinghai-Tibet Plateau within the southeast Tibet gorge forest area, it encompasses approximately 1.47 × 10^7^ hectares,^1^ accounting for 80% of Tibet's total forested area. The region experiences a climate characterized by an average annual temperature ranging from 6 °C to 17 °C, with less than 2100 hours annually. Solar radiation levels vary between 5460 and 7530 MJ/m², annual precipitation ranges from 500 to 1000 mm, relative humidity is between 60% and 75%, and wind speeds average 2 to 4 m/s. The climate is predominantly influenced by the Indian Ocean and the Pacific warm current. Meteorological data for Nyingchi city can be accessed online through the Nyingchi Municipal People's Government Network at <http://www.linzhi.gov.cn/linzhi/zmlz/qh.shtml>. Notably, the region lacks heavy industry, with tourism serving as the primary economic driver in the region.

**Untargeted metabolome measurements using ultra-high performance liquid chromatography - mass spectrometer (UHPLC-MS)**

Serum samples were preprocessed with slight modifications to previously described methods.^2^ Briefly, 100 μL of serum sample was transferred to an EP tube, followed by the addition of 300 μL of methanol containing 1 μg/mL L-2-chlorophenylalanine. The resulting mixture was vortexed for 30 seconds, sonicated for 10 minutes in an ice-water bath, and incubated for 1 h at -40 °C to precipitate proteins. Subsequently, the sample underwent centrifuged at 12000 rpm for 15 min at 4 °C. The resulting extract was then transferred to a clean glass vial for further analysis. A quality control (QC) sample was prepared by amalgamating equal aliquots of supernatants derived from each serum sample.

LC-MS/MS analyses were conducted utilizing a UHPLC 1290 system (Agilent Technologies, Waldbronn, Germany) coupled with a UPLC HSS T3 column (2.1 mm × 100 mm, 1.8 μm, Waters, Manchester, UK) and interfaced with a Q Exactive mass spectrometer (Orbitrap MS, Thermo Fisher Scientific, SanJose, CA). The extracts were gradient-eluted with water and acetonitrile with 0.1% formic acid for positive mode, or 5 mmol/L ammonium acetate for negative mode, and the mobile phase B was acetonitrile. The mass spectrometry was used for its ability to acquire MS/MS spectra on information-dependent acquisition (IDA) mode, controlled by Xcalibur 4.0.27 software (Thermo Fisher Scientific). The electrospray ionization (ESI) source conditions were configured as follows: sheath gas flow rate as 45 Arb, Aux gas flow rate as 15Arb, capillary temperature 400 °C, full MS resolution as 70000, MS/MS resolution as 17500, collision energy as 20/40/60 in NCE mode, spray Voltage as 4.0 kV (positive) or -3.6 kV (negative), respectively. To assess data repeatability, samples were randomized, and a QC sample was injected and analyzed after every 10 samples.^3^ The relative standard deviation (RSD) for internal standard in the QC samples averaged 7.97% in the analysis of human serum samples.

**Amino acids measurements using UHPLC-MS/MS**

Serum AAs quantification was performed as previously described with modifications.^4^ The serum samples were defrosted in an ice water bath and mixed in a vortex for 30 s. A precisely measured 50 μL aliquot from each sample was transferred to an Eppendorf tube. After the addition of 200 μL of extraction solution (acetonitrile-methanol, 1:1, with internal standard mixture (consisted of L-Tryptophan-(indole-d5), L-Methionine-13C,d3, 4-Aminobutyric acid-2,2,3,3,4,4-d6, L-Glutamic acid-13C5,15N, L-Serine-d3, L-Asparagic acid-d3, L-Arginine-13C6, L-Lysine-d4 HCl), precooled at -20 °C), the samples were vortexed for 30 s and sonicated for 15 min in ice-water bath, incubation at -40 °C for one hour, and centrifugation at 12000 rpm and 4 °C for 15 min. Then the supernatants were separated with Agilent 1290 Infinity II series UHPLC System (Agilent Technologies), equipped with a Waters ACQUITY UPLC BEH Amide column (100 × 2.1 mm, 1.7 μm, Waters). The column was eluted with acetonitrile-water solution containing 1% formic acid. The column temperature maintained at 35 °C. The auto-sampler temperature was set at 4 °C and the injection volume was 1 μL. MS analysis was carried out using Agilent 6460 triple quadrupole mass spectrometer (Agilent Technologies) equipped with an AJS electrospray ionization (AJS-ESI) interface. The typical ion source parameters included a capillary voltage of +4000/-3500 V, Nozzle Voltage of +500/-500 V, gas (N2) temperature = 300 °C, gas (N2) flow = 5 L/min, sheath gas (N2) temperature = 250 °C, sheath gas flow = 11 L/min, nebulizer = 45 psi. In the analysis of human serum samples, the RSD for internal standards mixture in the QC samples was on average of 5.97%, 7.30%, 8.23%, 10.03%, 8.27%, 17.67%, 7.57% and 8.70%, respectively.

**Short-chain fatty acids and medium-chain fatty acids measurements using the gas chromatography - mass spectrometer (GC-MS)**

Serum SCFAs and MCFAs quantification was performed as previously described.^5^ 50 μL serum was mixed with 0.05 mL 50 % H_2_SO_4_ and 0.2 mL of 2-Methylvaleric acid (25 mg/L stock in methyl tert-butyl ether) as internal standard. The amalgamation was subjected to vortex mixing for 30 s, oscillations in 10 min, then ultrasound treated for 10 min with incubation in ice water. Subsequently, centrifugation was conducted for 15 min at 10000rpm, 4 °C. Keep at −20 °C for 30 min, the organic phase was collected and analyzed using SHIMADZU GC2030-QP2020 NX gas chromatography-mass spectrometer (Shimadzu Corporation, Kyoto, Japan) equipped with a HP-FFAP capillary column (30m×250μm×0.25μm, Agilent Technologies, Wilmington, DE, USA). A 1 μL aliquot of the analyte was injected in split mode (5:1). Helium was used as the carrier gas, with a front inlet purge flow of 3 mL min^−1^, and a gas flow rate through the column was 1 mL min^−1^. The initial temperature was maintained at 80 °C for 1 min, subsequently elevated to 200 °C at a rate of 10 °C min^−1^ for 5 min, and then sustained for 1 min at 240 °C at a rate of 40 °C min^−1^. The injection, transfer line, quad and ion source temperatures were 240 °C, 240 °C, 200 °C and 150 °C, respectively. The energy was -70 eV in electron impact mode. The mass spectrometry data were acquired in Scan/SIM mode with the m/z range of 33-150 after a solvent delay of 3.5 min. The RSD for internal standard in the QC samples averaged at 6.24% during the analysis of human serum samples.

**Untargeted metabolomics data analysis**

The acquired MS data pretreatments included peak selection and grouping, retention time correction, second peak grouping, and isotopes and adducts annotation, which were performed as previously described with slight modifications.^6^ LC-MS raw data files were converted into mzXML format and subsequently analyzed using the XCMS and CAMERA toolbox with R statistical language. Employing retention time and the m/z data pairs as unique identifiers for each ion, we obtained ion intensities of each peak and generated a three-dimensional matrix containing arbitrarily assigned peak indices (retention time-m/z pairs), ion intensities (variables) and sample names (observations). Significant peaks were identified through comparison of exact molecular mass data (m/z) with entries in the Human Metabolome Database (HMDB) and KEGG database. Metabolites with a mass difference between observed and theoretical mass of less than 10 ppm were annotated, and the molecular formulas of matched metabolites were validated through isotopic distribution measurements. Verification and confirmation of metabolites were achieved using commercial reference standards, involving the comparison of MS/MS spectra and retention time. The matrix was further refined by removing peaks with missing values (ion intensity = 0) in more than 50% of samples and 20% of QC samples and those with isotope ions from each group to obtain consistent variables. Each retained peak was normalized to the QC sample using Robust Loess Signal Correction (R-LSC) based on the periodic analysis of the QC sample and the true samples to ensure the data of high quality within an analytical run, which is accepted as a quality assurance strategy in metabolic profiling. The relative s.d. (RSD) value of metabolites in the QC samples was set at a threshold of 30%, adhering to the standard for assessing repeatability in metabolomics datasets.

A linear mixed model, which considers repeated measurements from the same subjects at different time points, was conducted to investigate the relative abundance changes of metabolites over time using the ‘lme4’ package (v1.1). The model standardized the relative abundance of metabolites, with sample collection time designated as a fixed effect and subjects as random effects. *P* values underwent correction using the false discovery rate (fdr). An adjusted *P*-value less than 0.05 was considered statistically significant. Metabolomics Pathway Analysis (MetPA) was performed by online tool MetaboAnalyst with a hypergeometric test for over-representation analysis between and relative-between centrality for pathway topology analysis.^7^

**Serum, urine and liver biochemical analysis in mice**

The levels of urate and XOD were detected through commercial detection kits (Boxbio, AKAO014M and Jianglai, JL20379-96T, respectively) according to the instructions of manufacturers.

**Histological analysis in mice**

At the time of euthanasia, liver samples from the right lobe and kidney samples from the right side were collected. A portion of these sample was fixed in 4% paraformaldehyde, embedded in paraffin and stained with classical hematoxylin-eosin (H&E) for morphological assessment. Histological evaluations were analyzed using conventional light microscopy. After staining the tissue sections with H&E and properly mounting the slides, the slides were transferred to a light microscope for imaging.

**mRNA expression of liver and kidney tissues in mice**

mRNA was extracted from liver and kidney tissues using the FastPure Cell/Tissue Total RNA Isolation Kit V2 (Vazyme, RC112-01). cDNA was synthesized from the isolated mRNA using Hiscript III Reverse Transcriptase (Vazyme, R302-01). Quantitative PCR (qPCR) was performed with 40 cycles of denaturation at 95°C for 10 seconds, annealing at 60°C for 10 seconds, and extension at 70°C for 30 seconds, using ChamQ Universal SYBR qPCR Master Mix (Vazyme, Q711) and the primers listed in Table S13.^8^ All samples were analyzed in triplicate, and relative mRNA expression levels were calculated using the 2^–ΔΔCt^ method.

**Mice liver transcriptome sequencing**

Total RNA was extracted from the liver tissue using TRIzol® Reagent according to the manufacturer’s instructions. RNA quality was assessed using the 5300 Bioanalyzer (Agilent) and quantified with the ND-2000 spectrophotometer (NanoDrop Technologies). Only high-quality RNA samples (OD260/280 = 1.8–2.2, OD260/230 ≥ 2.0, RQN ≥ 6.5, 28S:18S ≥ 1.0, >1 μg) were used to construct the sequencing library. RNA purification, reverse transcription, library construction, and sequencing were performed at Shanghai Majorbio Bio-pharm Biotechnology Co., Ltd. (Shanghai, China) following the manufacturer’s instructions (Illumina, San Diego, CA). The RNA-seq transcriptome library was prepared using the Illumina® Stranded mRNA Prep, Ligation kit (Illumina, San Diego, CA) with 1 μg of total RNA. Briefly, messenger RNA was isolated using the polyA selection method with oligo(dT) beads and then fragmented using a fragmentation buffer. Double-stranded cDNA was synthesized using the SuperScript double-stranded cDNA synthesis kit (Invitrogen, CA) with random hexamer primers (Illumina). The synthesized cDNA underwent end-repair, phosphorylation, and 'A' base addition following Illumina’s library construction protocol. Libraries were size-selected for cDNA target fragments of 300 bp on 2% Low Range Ultra Agarose and then amplified by PCR using Phusion DNA polymerase (NEB) for 15 cycles. After quantification with Qubit 4.0, the paired-end RNA-seq library was sequenced on the NovaSeq X Plus sequencer (2 × 150 bp read length). The raw paired-end reads were trimmed and quality-controlled using fastp with default parameters.^9^ Clean reads were aligned to the reference genome in orientation mode using HISAT2 software.^10^ The mapped reads of each sample were assembled using StringTie in a reference-based approach.^11^

**RNA sequencing data analysis**

To identify differentially expressed genes (DEGs) between samples, transcript expression levels were calculated using the transcripts per million (TPM) method. Gene abundances were quantified using RSEM.^12^ Differential expression analysis was primarily conducted using DESeq2.^13, 14^ DEGs with |log2FC| ≥ 1 and adjusted *P*-value < 0.05 calculated by Benjamini & Hochberg (BH) method were considered to be significantly different expressed genes. Additionally, functional enrichment analyses, including Gene Ontology (GO) and Kyoto Encyclopedia of Genes and Genomes (KEGG) pathways, were performed to determine which DEGs were significantly enriched in GO terms and metabolic pathways compared to the whole-transcriptome background. GO functional enrichment analysis was performed using Goatools, and KEGG pathway analysis was conducted using Python's SciPy library. Gene set enrichment analysis based on the GO database was also performed, with *P*-value < 0.05 considered statistically significant.

**References**

(1) Wang, W.; Xu, W.; Wen, Z.; Wang, D.; Wang, S.; Zhang, Z.; Zhao, Y.; Liu, X., Characteristics of Atmospheric Reactive Nitrogen Deposition in Nyingchi City. *Scientific reports* **2019,** *9*, (1), 4645.

(2) Dunn, W. B.; Broadhurst, D.; Begley, P.; Zelena, E.; Francis-McIntyre, S.; Anderson, N.; Brown, M.; Knowles, J. D.; Halsall, A.; Haselden, J. N.; Nicholls, A. W.; Wilson, I. D.; Kell, D. B.; Goodacre, R., Procedures for large-scale metabolic profiling of serum and plasma using gas chromatography and liquid chromatography coupled to mass spectrometry. *Nature protocols* **2011,** *6*, (7), 1060-83.

(3) Contrepois, K.; Wu, S.; Moneghetti, K. J.; Hornburg, D.; Ahadi, S.; Tsai, M. S.; Metwally, A. A.; Wei, E.; Lee-McMullen, B.; Quijada, J. V.; Chen, S.; Christle, J. W.; Ellenberger, M.; Balliu, B.; Taylor, S.; Durrant, M. G.; Knowles, D. A.; Choudhry, H.; Ashland, M.; Bahmani, A.; Enslen, B.; Amsallem, M.; Kobayashi, Y.; Avina, M.; Perelman, D.; Schüssler-Fiorenza Rose, S. M.; Zhou, W.; Ashley, E. A.; Montgomery, S. B.; Chaib, H.; Haddad, F.; Snyder, M. P., Molecular Choreography of Acute Exercise. *Cell* **2020,** *181*, (5), 1112-1130.e16.

(4) Wang, Z.; Li, B.; Li, S.; Lin, W.; Wang, Z.; Wang, S.; Chen, W.; Shi, W.; Chen, T.; Zhou, H.; Yinwang, E.; Zhang, W.; Mou, H.; Chai, X.; Zhang, J.; Lu, Z.; Ye, Z., Metabolic control of CD47 expression through LAT2-mediated amino acid uptake promotes tumor immune evasion. *Nature communications* **2022,** *13*, (1), 6308.

(5) Hou, Y.; Zhang, Z.; Cui, Y.; Peng, C.; Fan, Y.; Tan, C.; Wang, Q.; Liu, Z.; Gong, J., Pu-erh tea and theabrownin ameliorate metabolic syndrome in mice via potential microbiota-gut-liver-brain interactions. *Food research international (Ottawa, Ont.)* **2022,** *162*, (Pt B), 112176.

(6) Liu, R.; Hong, J.; Xu, X.; Feng, Q.; Zhang, D.; Gu, Y.; Shi, J.; Zhao, S.; Liu, W.; Wang, X.; Xia, H.; Liu, Z.; Cui, B.; Liang, P.; Xi, L.; Jin, J.; Ying, X.; Wang, X.; Zhao, X.; Li, W.; Jia, H.; Lan, Z.; Li, F.; Wang, R.; Sun, Y.; Yang, M.; Shen, Y.; Jie, Z.; Li, J.; Chen, X.; Zhong, H.; Xie, H.; Zhang, Y.; Gu, W.; Deng, X.; Shen, B.; Xu, X.; Yang, H.; Xu, G.; Bi, Y.; Lai, S.; Wang, J.; Qi, L.; Madsen, L.; Wang, J.; Ning, G.; Kristiansen, K.; Wang, W., Gut microbiome and serum metabolome alterations in obesity and after weight-loss intervention. *Nature medicine* **2017,** *23*, (7), 859-868.

(7) Pang, Z.; Chong, J.; Zhou, G.; de Lima Morais, D. A.; Chang, L.; Barrette, M.; Gauthier, C.; Jacques, P.; Li, S.; Xia, J., MetaboAnalyst 5.0: narrowing the gap between raw spectra and functional insights. *Nucleic acids research* **2021,** *49*, (W1), W388-w396.

(8) Liu, J.; Gao, M.; He, J.; Wu, K.; Lin, S.; Jin, L.; Chen, Y.; Liu, H.; Shi, J.; Wang, X.; Chang, L.; Lin, Y.; Zhao, Y. L.; Zhang, X.; Zhang, M.; Luo, G. Z.; Wu, G.; Pei, D.; Wang, J.; Bao, X.; Chen, J., The RNA m(6)A reader YTHDC1 silences retrotransposons and guards ES cell identity. *Nature* **2021,** *591*, (7849), 322-326.

(9) Chen, S.; Zhou, Y.; Chen, Y.; Gu, J., fastp: an ultra-fast all-in-one FASTQ preprocessor. *Bioinformatics (Oxford, England)* **2018,** *34*, (17), i884-i890.

(10) Kim, D.; Langmead, B.; Salzberg, S. L., HISAT: a fast spliced aligner with low memory requirements. *Nature methods* **2015,** *12*, (4), 357-60.

(11) Pertea, M.; Pertea, G. M.; Antonescu, C. M.; Chang, T. C.; Mendell, J. T.; Salzberg, S. L., StringTie enables improved reconstruction of a transcriptome from RNA-seq reads. *Nature biotechnology* **2015,** *33*, (3), 290-5.

(12) Li, B.; Dewey, C. N., RSEM: accurate transcript quantification from RNA-Seq data with or without a reference genome. *BMC bioinformatics* **2011,** *12*, 323.

(13) Love, M. I.; Huber, W.; Anders, S., Moderated estimation of fold change and dispersion for RNA-seq data with DESeq2. *Genome biology* **2014,** *15*, (12), 550.

(14) Wang, L.; Feng, Z.; Wang, X.; Wang, X.; Zhang, X., DEGseq: an R package for identifying differentially expressed genes from RNA-seq data. *Bioinformatics (Oxford, England)* **2010,** *26*, (1), 136-8.
