# Supplementary material for: Serum metabolomic profiling uncovered metabolic shifts in individuals upon moderate-altitude exposure and identified the potentiality of beta-alanine to ameliorate hyperuricemia
Source: Redox Biol. 2025 Feb 28;81:103546. doi: 10.1016/j.redox.2025.103546 (PMC11930757; doi:10.1016/j.redox.2025.103546)

**A**

| | Internal standards | Relative standard deviation (RSD) |
|---|---|---|
| Untargeted metabolome profiles | L-2-chlorophenylalanine | 7.97% |
| Targeted AAs profiles | L-Tryptophan-(indole-d5) | 5.97% |
| | L-Methionine-13C,d3 | 7.30% |
| | 4-Aminobutyric acid-2,2,3,3,4,4-d6 | 8.23% |
| | L-Glutamic acid-13C5,N15 | 10.03% |
| | L-Serine-d3 | 8.27% |
| | L-Asparagic Acid-d3 | 17.67% |
| | L-Arginine-13C6 | 7.57% |
| | L-Lysine-d4 HCl | 8.70% |
| Targeted SCFAs and MCFAs profiles | 2-Methylvaleric acid | 6.24% |

**B**

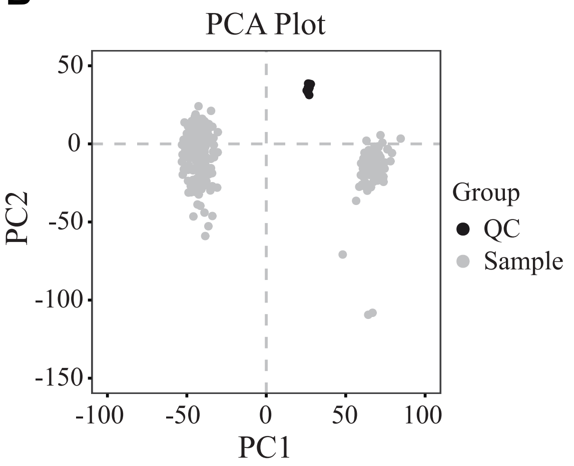


**C**

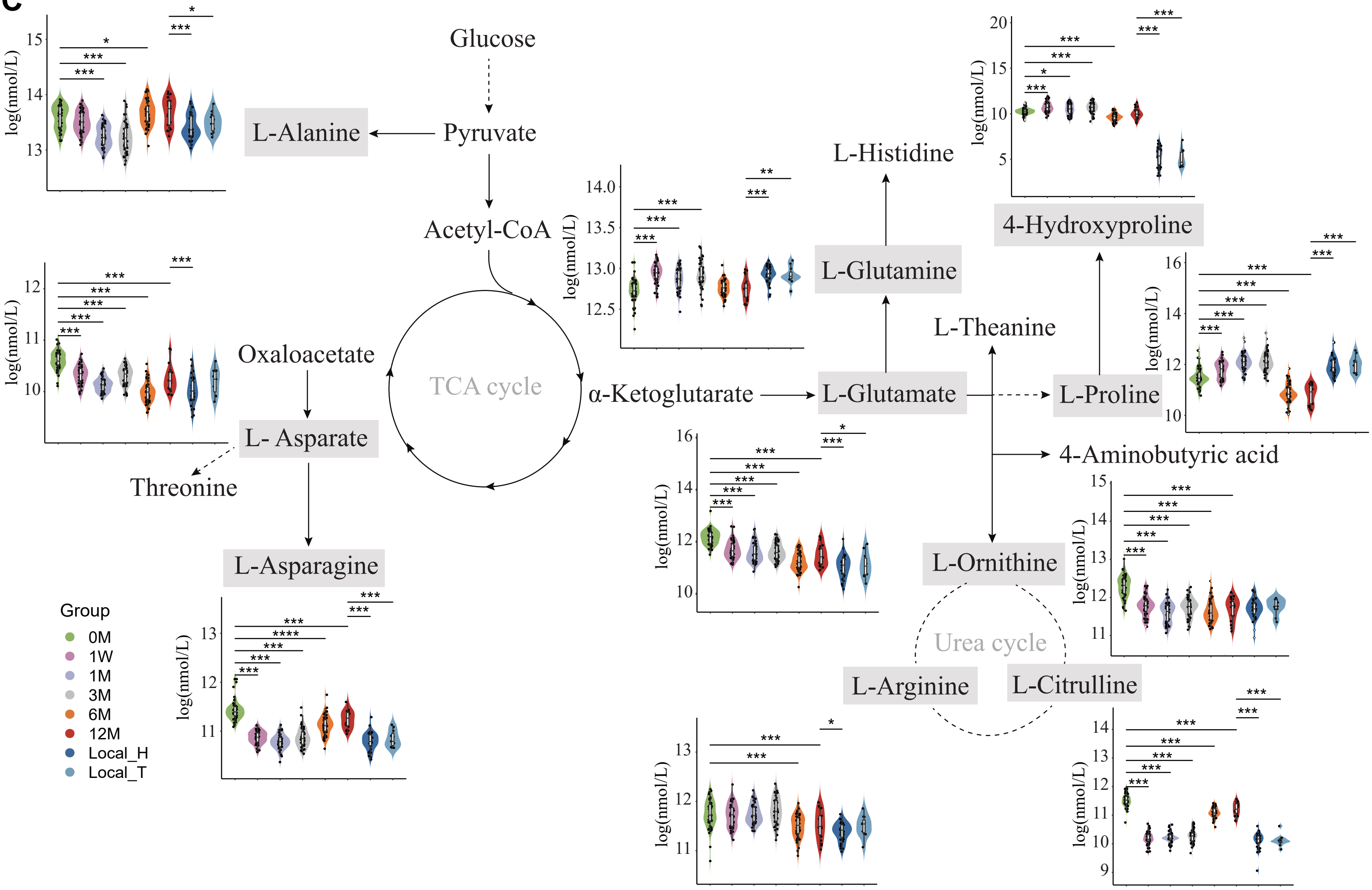

Supplement: Fig. S1 — Quality control analysis in serum metabolome measurement and an atlas of L-Arginine engaged metabolism pathways associated with the healthy individuals exposed to moderate altitude. A The relative standard deviations of internal standards in untargeted metabolome and targeted metabolome (amino acids, AAs; short-chain fatty acids, SCFAs; medium-chain fatty acids, MCFAs) analysis. B The principal component analysis (PCA) scores plot for quality control (QC) samples in untargeted metabolomics profile. C Amino acids (AAs) associated with L-Arginine metabolism, such as Arginine biosynthesis pathway, Arginine and Proline metabolism, and Alanine, Aspartate, and Glutamate metabolism, were colored with grey. Plotted are interquartile ranges (IQRs; boxes), medians (dark lines in the boxes), the lowest and highest values within 1.5 times IQR from the first and third quartiles (lines above and below the boxes), and density of values (width between curves). P-value of paired/unpaired samples was calculated with paired/unpaired two-tailed wilcox test. ∗P < 0.05; ∗∗P < 0.01; ∗∗∗P < 0.001; NS, not significant. [file mmc3.pdf]
