# Supplementary figures and images for: Serum metabolomic profiling uncovered metabolic shifts in individuals upon moderate-altitude exposure and identified the potentiality of beta-alanine to ameliorate hyperuricemia
Source: Redox Biol. 2025 Feb 28;81:103546. doi: 10.1016/j.redox.2025.103546 (PMC11930757; doi:10.1016/j.redox.2025.103546)

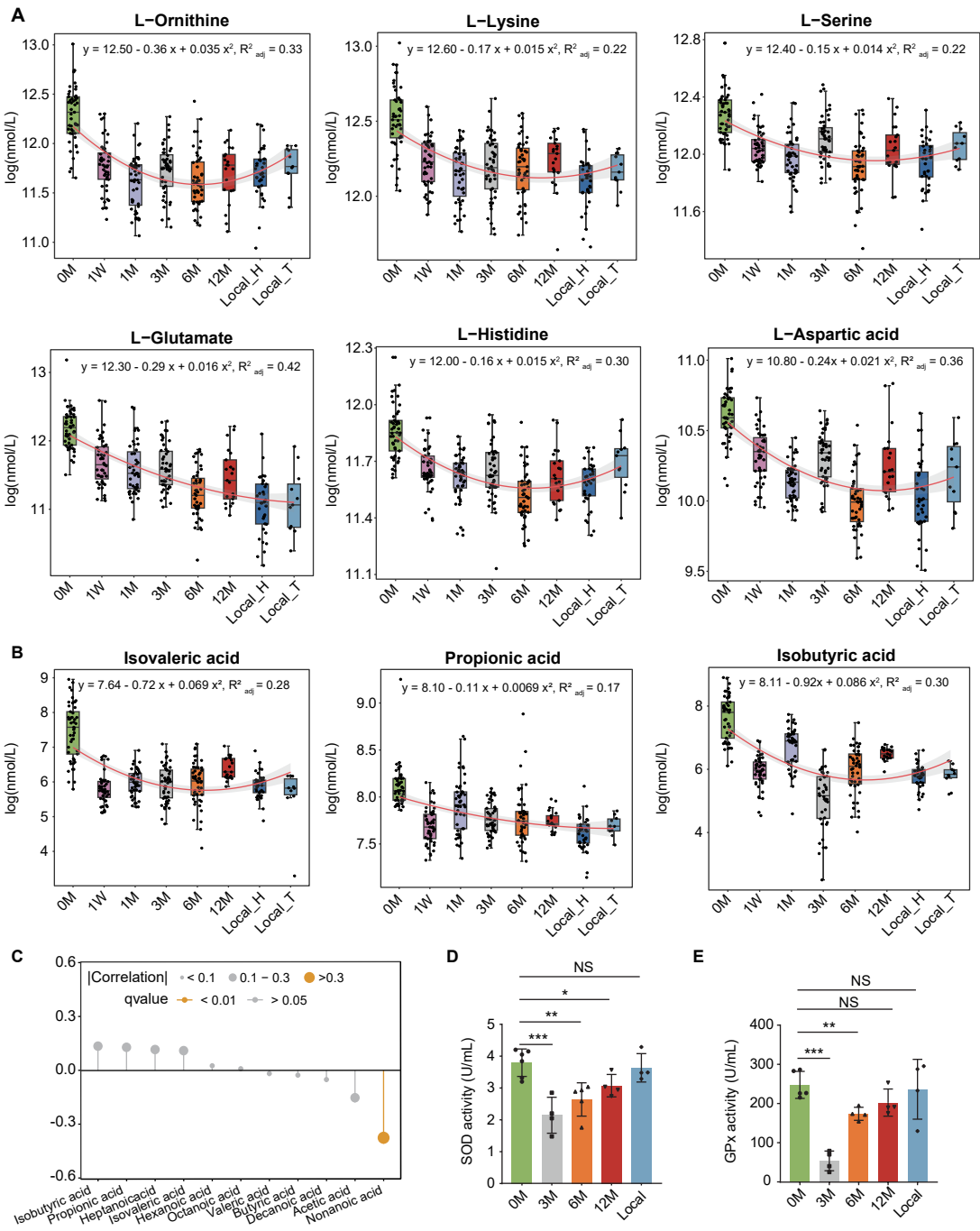

Supplement: Fig. S2 — Trajectories of metabolites significantly associated with serum urate and correlation analysis between Beta-Alanine and fatty acids, and oxidative stress indexes measurements. A Boxplot of amino acids significantly correlated with serum urate. Plotted are interquartile ranges (IQRs; boxes), medians (dark lines in the boxes), the lowest and highest values within 1.5 times IQR from the first and third quartiles (lines above and below the boxes), and outliers beyond the lines (circles). B Boxplot of short-chain fatty acids and medium-chain fatty acids significantly correlated with serum urate. Plotted as A. C Rmcorr correlation analysis of Beta-Alanine and fatty acids. The size and color of each circle are based on Rmcorr correlation coefficients and adjusted Rmcorr P-value, respectively. D-E Superoxide Dismutase (SOD) and Glutathione Peroxidase (GPX) levels in individuals before and after moderate-altitude exposure. Data were expressed as mean ± SE. P-value of paired/unpaired samples was calculated with paired/unpaired two-tailed wilcox test. ∗P < 0.05; ∗∗P < 0.01; ∗∗∗P < 0.001. [file mmc4.pdf]
