# Supplementary material for: Serum metabolomic profiling uncovered metabolic shifts in individuals upon moderate-altitude exposure and identified the potentiality of beta-alanine to ameliorate hyperuricemia
Source: Redox Biol. 2025 Feb 28;81:103546. doi: 10.1016/j.redox.2025.103546 (PMC11930757; doi:10.1016/j.redox.2025.103546)
Supplement: Fig. S3 — Violin plot of literatures reported gut microbiota engaged in the metabolism of urate in another cohort. A Relative abundance of gut microbiota at phylum, genus and species levels related to urate metabolism. B Relative abundance of K01918 in the metabolism of Beta-Alanine to anti-hyperuricemic anserine. Plotted are interquartile ranges (IQRs; boxes), medians (dark lines in the boxes), the lowest and highest values within 1.5 times IQR from the first and third quartiles (lines above and below the boxes), and density of values (width between curves). P-value of paired/unpaired samples was calculated with paired/unpaired two-tailed wilcox test. ∗∗P < 0.01; ∗P < 0.05; +P < 0.1; NS, not significant. [file mmc5.pdf]

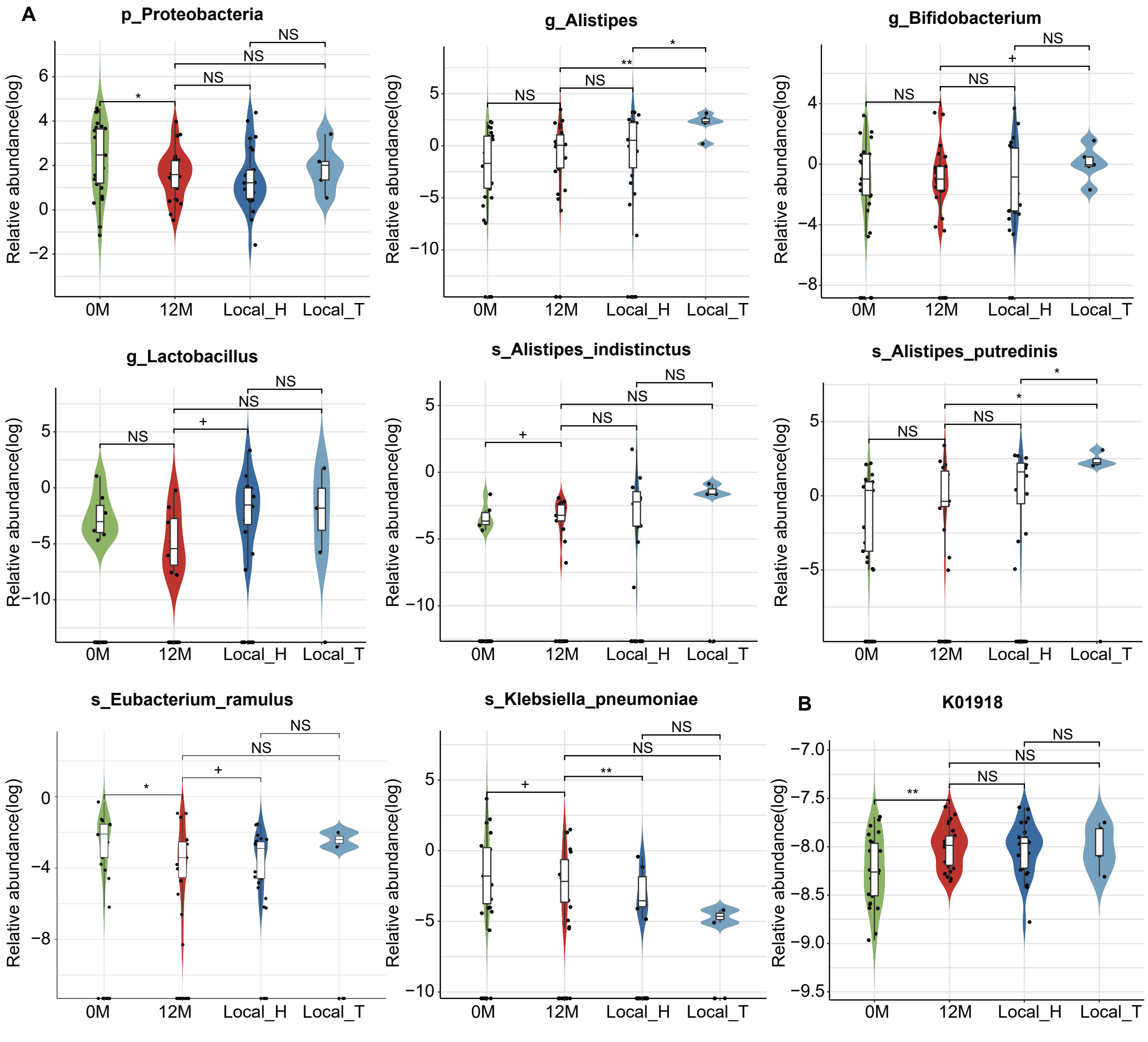
A
p_Proteobacteria
g_Alistipes
g_Bifidobacterium
g_Lactobacillus
s_Alistipes_indistinctus
s_Alistipes_putredinis
s_Eubacterium_ramulus
s_Klebsiella_pneumoniae
B
K01918
Relative abundance(log)
0M
12M
Local_H
Local_T
NS
*
**
+
